# Elucidating the Role of the Metal Catalyst and Oxide Support in the Ru/CeO_2_-Catalyzed CO_2_ Methanation Mechanism

**DOI:** 10.1021/acs.jpcc.1c07537

**Published:** 2021-11-16

**Authors:** Sergio López-Rodríguez, Arantxa Davó-Quiñonero, Esther Bailón-García, Dolores Lozano-Castelló, Facundo C. Herrera, Eric Pellegrin, Carlos Escudero, Max García-Melchor, Agustín Bueno-López

**Affiliations:** † Departamento de Química Inorgánica, Universidad de Alicante, Carretera San Vicente del Raspeig s/n, E-03080 Alicante, Spain; ‡ School of Chemistry, CRANN and AMBER Research Centres, Trinity College Dublin, College Green, Dublin 2, Ireland; § ALBA Synchrotron Light Source, Carrer de la Llum 2-26, Cerdanyola del Vallès, 08290 Barcelona, Spain; ∥ Instituto de Investigaciones Fisicoquímicas Teóricas y Aplicadas (INIFTA, CONICET), Departamento de Química, Facultad de Ciencias Exactas, Universidad Nacional de La Plata, Diagonal 113 y 64, 1900 La Plata, Argentina

## Abstract

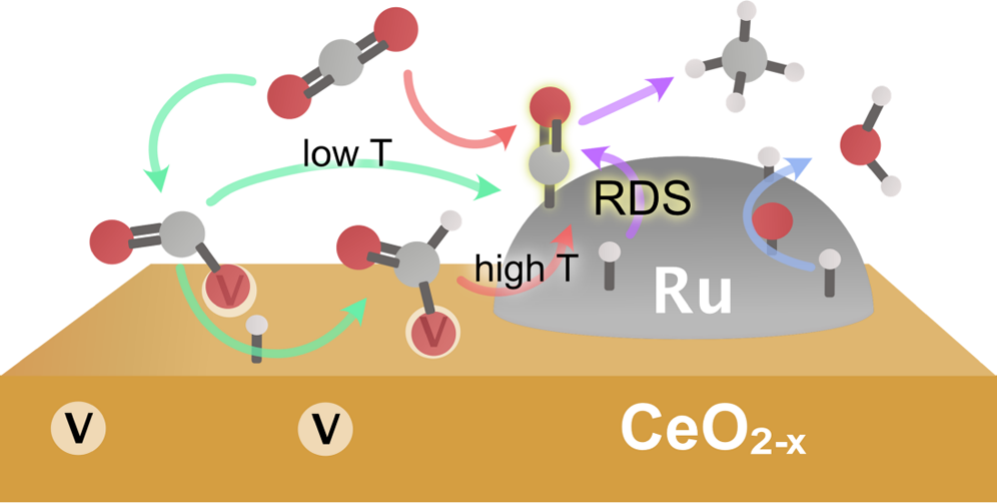

This study addresses the yet unresolved CO_2_ methanation mechanism on a Ru/CeO_2_ catalyst by means of near-ambient-pressure X-ray photoelectron spectroscopy (NAP–XPS) and diffuse reflectance infrared Fourier transform spectroscopy (DRIFTS) complemented with periodic density functional theory (DFT) calculations. NAP–XPS results show that the switch from H_2_ to CO_2_ + H_2_ mixture oxidizes both the Ru and CeO_2_ phases at low temperatures, which is explained by the CO_2_ adsorption modes assessed by means of DFT on each representative surface. CO_2_ adsorption on Ru is dissociative and moderately endergonic, leading to polybonded Ru-carbonyl groups whose hydrogenation is the rate-determining step in the overall process. Unlike on Ru metal, CO_2_ can be strongly adsorbed as carbonates on ceria surface oxygen sites or on the reduced ceria at oxygen vacancies as carboxylates (CO_2_^–δ^), resulting in the reoxidation of ceria. Carboxylates can then evolve as CO, which is released either via direct splitting at relatively low temperatures or through stable formate species at higher temperatures. DRIFTS confirm the great stability of formates, whose depletion relates with CO_2_ conversion in the reaction cell, while carbonates remain on the surface up to higher temperatures. CO generation on ceria serves as an additional reservoir of Ru-carbonyls, cooperating to the overall CO_2_ methanation process. Altogether, this study highlights the noninnocent role of the ceria support in the performance of Ru/CeO_2_ toward CO_2_ methanation.

## 1 Introduction

The global warming induced by the rising energy-related CO_2_ emissions is a threat that has become a primary focus for environmental research. Moreover, according to official forecasts, the energy demand will grow by 33% in 2050 compared to 2017 together with the CO_2_ emissions over the next years.^1^ Hence, supplying this demand while meeting the CO_2_ emission targets is a major challenge that society has to face in the 21st century via the implementation of sustainable policies and energy practices with a lower carbon footprint.^2^ In this scenario, scientists and industrial stakeholders are urged to design alternative technologies to enable a feasible and successful transition from carbon-based to renewable fuels. This involves the deployment of new hydrogen power plants and the reduction of carbon dioxide emissions according to the established pillars of the circular economy.^3,4^ In this regard, the CO_2_ methanation reaction is an attractive strategy.^5,6^ This process consists in the conversion of CO_2_ to CH_4_, a valuable fuel so-called synthetic natural gas, using hydrogen as shown in eq 1
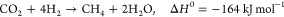
1As a remarkable positive incentive, CO_2_ methanation technologies based on captured CO_2_ and green hydrogen^7^ can enable a clean energy distribution without the requirement of new infrastructure or alternative combustion engines.^8^ Furthermore, CO_2_ methanation can be deemed carbon-neutral if both hydrogen and the thermal activation energy required are supplied from renewable sources and the CO_2_ released upon the eventual CH_4_ utilization is captured and fed back into the methanation process.

In general terms, CO_2_ methanation is spontaneous at ambient temperature, but the use of active catalysts is needed to overcome the sluggish reaction kinetics to reach relevant reaction rates and selectivities for industrial applications.^9^ Among the studied active phases, nickel is a well-known cost-effective CO_2_ methanation catalyst that operates between 300 and 450 °C providing good CH_4_ selectivity. When finely dispersed as Ni nanoparticles, the choice of the support determines the overall performance. For example, literature reports have shown that CO_2_ Ni/CeO_2_ formulations are more active than their counterparts with TiO_2_, Al_2_O_3_, and ZrO_2_.^10−17^ Such improved performance is generally attributed to the well-known oxygen storage capacity and oxygen mobility in ceria.^10,18−21^ Yet, Ni catalysts suffer from sintering and the reverse water gas-shift reaction (RWGS) at high operating temperatures, which compromises CH_4_ selectivity and CO_2_ conversion.

On the other hand, noble-metal-based catalysts such as Rh, Pt, Pd, and Ru^22−27^ exhibit a lower CO_2_ methanation onset than Ni (from *ca*. 250 °C to *ca*. 180 °C) enabling us to operate at temperatures where the RWGS is rendered thermodynamically unfavorable. Among precious metals, Ru has received great attention^28−36^ and the CO_2_ methanation mechanism on this metal catalyst has been investigated in detail.^30,36,37^ However, many important aspects of the underlying mechanism still remain unclear and no general consensus has been reached yet, mainly due to the strong structure-sensitive nature of this reaction.^24,38,39^ On the one hand, many studies have reported plausible reaction pathways featuring formates (*OCHO, where * denotes a surface-active site) as key intermediates formed upon CO_2_ adsorption on H-terminated Ru surfaces. According to this approach, further hydrogenation steps from formates using chemisorbed *H would give rise to *C*_x_*H*_y_*O intermediate species up to methane.^19,22,35,40,41^ On the other hand, alternative mechanisms for the Ru-catalyzed methanation have been proposed by different authors. Proaño et al.^41^ postulated that Ru-carbonyls are direct precursors of CH_4_ on Ru/TiO_2_ catalysts. On the contrary, Falbo et al.^30^ proposed a mechanism for Ru/Al_2_O_3_, wherein CO_2_ is adsorbed as a bicarbonate on the support and subsequently hydrogenated to CH_4_ on Ru, involving formate and carbonyl intermediates. For Ru/CeO_2_, Wang et al.^42,43^ reported that CO_2_ methanation involves formate intermediates, which dissociate on the vacancy sites in ceria. Despite all of these insights, the detailed roles of ruthenium, the ceria support, and the interfacial sites remain unclear. Similarly to Ni, CeO_2_ and CeO_2_-modified supports provide enhanced activity for Ru catalysts,^29,44,45^ although the nature of their active sites is not completely understood either.

Herein, we report a thorough mechanistic investigation of the CO_2_ methanation reaction on a high-performance Ru/CeO_2_ catalyst using advanced spectroscopy techniques and periodic density functional theory (DFT) calculations. The changes in the electronic structure of Ru and Ce were monitored under reaction conditions using synchrotron radiation by *in situ* near-ambient-pressure X-ray photoelectron spectroscopy (NAP–XPS). DFT calculations, together with *in situ* diffuse reflectance infrared Fourier transform (DRIFT) spectroscopy experiments, were used to identify the active sites and the role of the chemisorbed species during reaction. Altogether, this multidisciplinary study demonstrates that CO_2_ is activated on ruthenium, leading to strongly bound carbonyl species, while the main role of ceria is to promote CO_2_ chemisorption in the form of carbonates and carboxylates. Further activation of CO_2_ on ceria is found to proceed via the decomposition of formate species on surface oxygen vacancies in complementary action to ruthenium. DFT calculations also indicate that the hydrogenation of Ru-carbonyls is the rate-determining step (RDS), while the subsequent hydrogenation of CO*_x_*H intermediates is thermodynamically driven.

## 2 Materials and Methods

### 2.1 Catalyst Synthesis and Characterization

Cerium oxide support was obtained by calcination of Ce(NO_3_)_3_·6H_2_O (99.0%, Sigma-Aldrich) for 6 h at 600 °C. Ruthenium was loaded by incipient wetness impregnation of ruthenium(III) acetylacetonate (97.0%, Sigma-Aldrich) dissolved in toluene to achieve a nominal 4% w. Finally, to obtain the Ru/CeO_2_ catalyst, the impregnated sample was heated under N_2_ atmosphere at a 5 °C/min pace up to 350 °C and was kept at this temperature for 3 h. General characterization results are presented in the Supporting Information, including N_2_ adsorption–desorption isotherms (Table S1, Figure S1), X-ray diffraction (Figure S2), and temperature-programmed reduction with H_2_ (Figure S3).

### 2.2 CO_2_ Methanation Catalytic Tests

CO_2_ methanation activity tests were performed in a fixed-bed tubular reactor (10 mm inner diameter) containing 200 mg of catalyst mixed with SiC particles (1.00–1.25 mm) to reach the bed volume of 1 cm^3^. The catalyst was pretreated *in situ* at 550 °C for 1 h under 200 mL/min of a 50% H_2_/N_2_ mixture. Once this pretreatment was completed, and after cooling down to 100 °C, the reaction mixture was introduced into the reactor. The feed consisted of 200 mL/min of 16% CO_2_, 64% H_2_, and 20% N_2_ at atmospheric pressure. The GHSV was 9000 h^–1^, and the temperature was increased in steps of 100 °C up to 325 °C. The gas composition was monitored under steady-state conditions at each temperature with specific gas analyzers (AwiteFLEX COOL; NDIR, electrochemical and thermal conductivity detectors) for CO, CO_2_, CH_4_, O_2_, and H_2_. A cold trap at −96 °C is placed after the reactor to condense the released water vapor prior to the detectors. The measured outlet concentrations are rescaled to keep the mol balance.

### 2.3 *In Situ* NAP–XPS Experiments

X-ray photoemission spectra were recorded *in situ* under methanation conditions at the near-ambient-pressure photoemission (NAPP) branch from CIRCE beamline at the ALBA Synchrotron Light Source facility,^46^ which allows tuning the photon energy within 100–2000 eV using a PHOIBOS NAP150 energy analyzer (SPECS GmbH). In each experiment, samples were exposed in the analysis chamber to the reaction mixture consisting of 16% CO_2_, 64% H_2_, and 20% N_2_. Two photon energies were used for each region of interest to obtain information at different surface depths, namely, 1372 and 1082 eV for the Ce 3d region, and 972 and 722 eV for the Ru 3d region. The inelastic mean free path for the emitted photoelectrons through the different pure solid phases of Ru and Ce at each energy utilized during the experiments is presented in Table S3.^47^

The Ru/CeO_2_ sample was pelletized and mounted on a gold mesh to minimize charging during the measurements and to provide an energy reference (Au 4f_7/2_ peak at 84.0 eV). An infrared laser system (808 nm) was used to heat the samples while the temperature was monitored using a K-type thermocouple. The pressure in the analysis chamber was kept at 1 mbar during the reaction. The catalyst was pretreated *in situ* at 550 °C for 1 h with 50% H_2_/N_2_ and then cooled down to 100 °C. After this procedure, the methanation mixture was fed at 30 mL/min and the temperature was increased in steps of 50 °C until 450 °C. The reaction was monitored with a mass spectrometer (MKS Instruments) installed at the second stage of the differential pumping system of the analyzer, and XPS measurements were recorded at each temperature under steady-state conditions.

### 2.4 *In Situ* DRIFTS Experiments

*In situ* DRIFTS experiments were performed in a Jasco infrared spectrometer, model FT/IR-4000, a Praying Mantis high-temperature reaction chamber (Harrick Scientific) with temperature and gas flow control. DRIFT spectra were recorded using a thermoelectrically liquid nitrogen-cooled photoconductive HgCdTe (mercury cadmium telluride, MCT) detector. The gas composition was monitored during the experiments with a Pfeiffer Vacuum mass spectrometer (OmniStar). The catalytic bed consisted of 90 mg of catalyst, which was pretreated in 50% H_2_/He at 450 °C for 1 h and then cooled down to room temperature under He atmosphere. A background spectrum was recorded under these conditions, and then the methanation mixture (10% CO_2_, 40% H_2_ in He balance) was fed at 100 mL/min. Spectra were recorded from 4000 to 1000 cm^–1^ at 25, 100, 200, 350, 400, and 450 °C once steady-state conditions were reached.

### 2.5 Computational Methods

Periodic DFT calculations were performed using the Perdew–Burke–Ernzenhof (PBE) exchange-correlation functional^48^ as implemented in the Vienna Ab initio Simulation Package (VASP) code, version 5.4.4.^49,50^ Projector augmented wave (PAW) potentials^51^ were used to describe the core electrons of Ce, Ru, O, C, and H ions, while plane waves with a kinetic cutoff energy of 500 eV were employed to represent their valence electrons. For Ce atoms, an additional on-site correction for the electrons localized in the 4f orbital was introduced using an effective Hubbard *U* term (*U*_eff_) of 4.5 eV following Dudarev’s approach.^52^

The CeO_2_ and metal Ru metal bulk structures were optimized with a Γ-centered Monkhorst–Pack k-point grid of 7 × 7 × 7 and 11 × 11 × 11, respectively, and the equilibrium lattice parameter was calculated using the Birch–Murnaghan equation of state. The most stable facets, *i.e*., CeO_2_(111) and Ru(0001),^53,54^ were modeled as surface slabs with at least a 15 Å vacuum gap in the perpendicular direction to the surface to avoid the interaction between the top and bottom layers. The CeO_2_(111) and Ru(0001) slabs were built with a periodicity of *p*(2 × 2), consisting of three and four metal layers, respectively. In both cases, the two upper layers were allowed to relax optimizing the structure using a k-point mesh of 3 × 3 × 1 and 7 × 7 × 1, respectively. The CeO_2_(111) and Ru(0001) slabs were constituted by three and four metal layers, respectively. Once optimized, the adsorption energies, Δ*E*_ads_, of the CO_2_ methanation intermediates were computed as
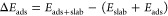
2where *E*_slab_ is the energy of the clean slab, *E*_ads_ is the energy of the adsorbate species in the gas phase, and *E*_ads+slab_ is the energy of the slab with the specific adsorbates in the most favorable configuration. These values were employed to calculate the Gibbs adsorption energies, Δ*G*_ads_, using the following equation
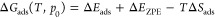
3where Δ*E*_ZPE_ and *T*Δ*S*_ads_ denote the changes in zero-point energy and entropy, respectively, relative to the clean slab and the adsorbate molecules in the gas phase. The effect of hydrogen and CO_2_ partial pressures on the above Gibbs adsorption energies was introduced as follows
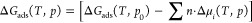
4where Δμ*_i_*(*T*, *p*) is the change in chemical potential of the *n* adsorbed species *i* at a given temperature and pressure, defined as
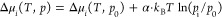
5where *k*_B_ is the Boltzmann constant, α is taken as 1/2 for H_2_ and 1 for CO_2_, and Δμ*_i_*(*T*, *p*_0_) is the change in chemical potential for H_2_ or CO_2_ at a given temperature and standard pressure.

After assessing the resting state of the Ru(0001) and CeO*_x_*(111) surfaces in the representative thermodynamic reactions conditions, the CO_2_ mechanism was investigated by computing the relative energies of the most plausible reaction intermediates on each surface, leading to different reaction pathways. Transition states (TS) for the relevant steps were located by means of the climbing image nudge elastic band method (CI-NEB) using at least five images along the reaction coordinate and the limited-memory Broyden–Fletcher–Goldfarb–Shanno (LBFGS) optimizer. The nature of TSs obtained was verified via vibrational frequency analysis using the finite difference method with a displacement of 0.01 Å.

## 3 Results and Discussion

### 3.1 Catalytic Activity

**Figure 1:**
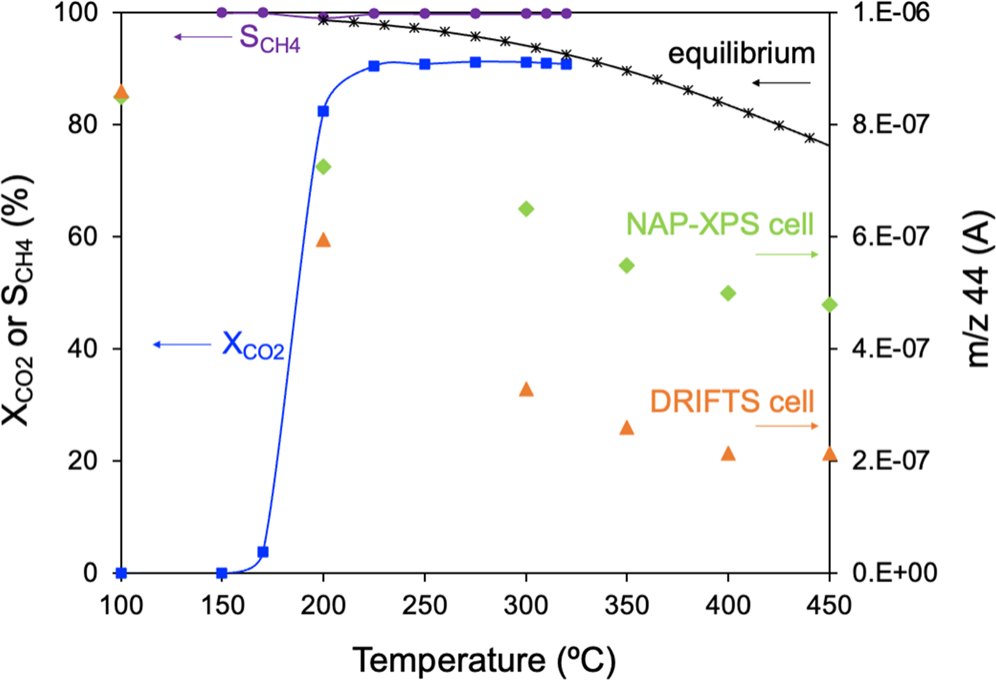
Primary *y*-axis: CO_2_ conversion (*X*_CO2_, squares) and CH_4_ selectivity (*S*_CH4_, circles) as a function of the temperature performed in a fixed-bed conventional reactor. Secondary *y*-axis: *m*/*z* 44 signal measured by mass spectrometry during DRIFTS (triangles) and NAP–XPS experiments (diamonds).

CO_2_ methanation tests were conducted in a conventional fixed-bed reactor containing the Ru/CeO_2_ catalyst, and the evolution of CO_2_ and CH_4_ gases produced during the reaction was monitored as shown in Figure 1. Prior to the experiment, the catalyst was pretreated with H_2_ at 550 °C for 1 h to reduce the ruthenium species present, in agreement with H_2_-TPR profiles (Figure S3). In this experimental setup, the CO_2_ methanation activity starts above 170 °C showing a sharp increase in the CO_2_ conversion (*X*_CO2_) with temperature until a stationary state is achieved at 225 °C. This behavior is similar to that reported in the literature for other Ru/CeO_2_ catalysts.^42,43^ Notably, only CH_4_ and H_2_O were detected as reaction products regardless of the temperature, which demonstrates the high selectivity toward CH_4_ relative to other byproducts such as CO. The *m*/*z* 44 signal monitored by mass spectrometry during the *in situ* DRIFTS and *in situ* NAP–XPS experiments under CO_2_ methanation reaction conditions is also included in Figure 1 for comparison. CO_2_ conversion displays similar onsets in the three experimental setups (*ca*. 170 °C), but the effect of temperature on CO_2_ consumption is different for each technique. The comparison of the catalytic behavior in the different experimental setups is essential to obtain a meaningful interpretation of experimental results since the reaction conditions used in the conventional fixed-bed reactor are different from those used in spectroscopic experiments (DRIFTS and NAP–XPS) due to the restrictions imposed by the spectroscopy setups.

### 3.2 *In Situ* DRIFTS Experiments

**Figure 2:**
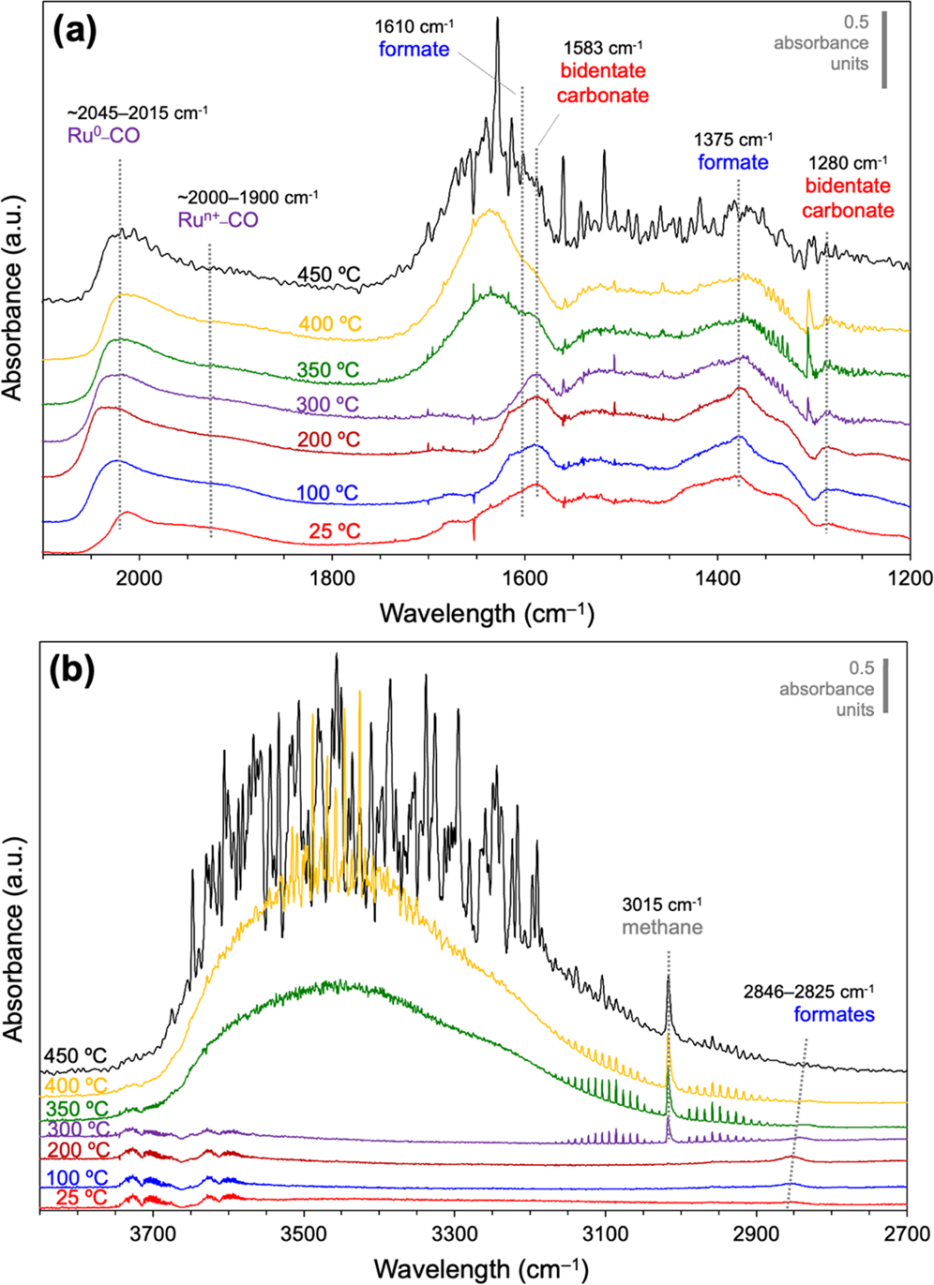
*In situ* DRIFT spectra recorded in steady state under CO_2_ methanation gas mixture (CO_2_/H_2_ 1:4) after a reduction pretreatment at 450 °C in H_2_/He in the regions: (a) 2100–1200 cm^–1^ and (b) 3800–2700 cm^–1^.

Figure 2 shows two relevant wavelength ranges selected from the spectra recorded after steady state was achieved at different temperatures. Figure 2a plots the 2100–1200 cm^–1^ range, which reveals the characteristic bands assigned to different vibration modes of C–O and C=O bonds, where formates (*OCHO) and carbonates (*OCOO) can be discerned, besides the formation of molecular H_2_O. The band centered at *ca*. 2000 cm^–1^ is attributed to Ru-carbonyls, showing a lower-wavenumber tail characteristic of their multiple configurations. The linear Ru^0^–CO shows between 2045 and 2015 cm^–1^, while oxidized Ru^*n*+^–CO appears at lower wavenumbers.^55^ This assignment contradicts what one would expect due to the lower back-donation from cationic species, but has been elsewhere reported and attributed to lower adsorbate–adsorbate interactions in the oxidized environment.^56^ Regarding this, as precisely detailed by Solís-García et al. in a recent paper, we recognize that there is, unfortunately, a large variety and contradictory band assignments on Ru carbonyls in the literature.^57^ For instance, according to some studies, bridged zerovalent Ru carbonyls may also be contributing to the broad carbonyl band in the lower-wavenumber region (*i.e*., 2000–1900 cm^–1^),^58^ although these species have been more frequently reported at *ca*. 1800 cm^–1^.^59,60^ The metallic linear carbonyls display a reversible blueshift to 2045 cm^–1^ attributed to the reduction of their oxidized surface environment by the effect of the reaction mixture followed by a decrease of CO coverage above the methanation onset, setting the Ru^0^–CO band back to 2015 cm^–1^. On the other hand, we do not see a sign of polybonded carbonyls either on metal or cationic Ru sites.

Figure 2b, displaying the 3800–2700 cm^–1^ range, provides information about C–H bonds, including the fingerprint bands of formates, gas-phase CH_4_, and O–H vibration bands assigned to surface hydroxyls and water.

Figure 2 proves that CO_2_ is chemisorbed on the reduced catalyst at room temperature, as seen by the formation of bidentate carbonates (1583 and 1280 cm^–1^ bands), formates (2846, 1610, and 1375 cm^–1^ bands), and Ru-carbonyls. Carbonates are formed upon CO_2_ chemisorption on metal oxides and are assumed to sit on the ceria surface as ruthenium is mostly reduced according to H_2_-TPR experiments (Figure S3). Notably, the presence of formates at 25 °C indicates that partial hydrogenation of the chemisorbed CO_2_ takes place at room temperature. The H atom in the formate species (*OCHO) can be sourced from either the dissociated H_2_ on the catalyst after reduction at 450 °C, presumably on Ru^0^, or the hydroxyl groups formed on ceria.^61,62^ Isolated hydroxyls are observed in the 25–300 °C range in the spectra as weak bands in the 3750–3600 cm^–1^ region, which are eventually masked below the broad and intense absorption band of polybonded OH or surface H_2_O. Since the consumption of hydroxyls is not detected, the first hypothesis regarding the generation of formates is more likely. We also note that the formation of Ru-carbonyls involves the dissociation of the CO_2_ molecule, which occurs on the reduced catalyst even at room temperature, despite the methanation reaction onset in this experimental setup is only observed at higher temperatures (see Figure 1). From the DRIFT spectra, however, it is not possible to discern whether CO_2_ splits directly onto the Ru particles or the CeO_2_ surface followed by CO spillover to the Ru phase. As stated above, the carbonyl band shape allows us to infer the coexistence of both on Ru^0^ and on Ru^*n*+^ species, although Ru is reduced after H_2_ pretreatment. The identification of carbonyls on cationic ruthenium (Ru^*n*+^–CO) could be evidence of the partial oxidation of ruthenium upon CO_2_ exposure at room temperature. This oxidation can be the result of the direct dissociative CO_2_ chemisorption into oxygen atoms and carbonyls. However, this is just a hypothesis since the effect of ceria in keeping Ru oxidized through synergistic interactions cannot be ruled out.

**Figure 3:**
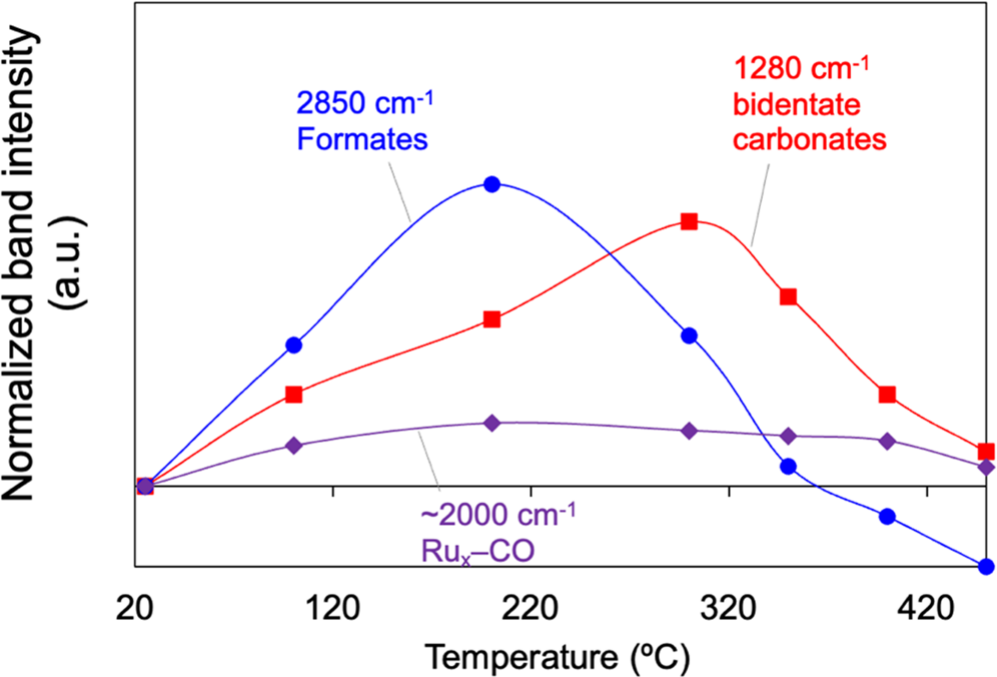
Evolution of formates, bidentate carbonates, and ruthenium carbonyl signals as a function of temperature for the experiments performed in the DRIFTS reactor cell under CO_2_ methanation gas mixture (10% CO_2_, 40% H_2_, 50% He) after a reduction pretreatment at 450 °C with 50% H_2_/He.

Interestingly, we observed that the increase in temperature has an effect on the intensity of the DRIFTS signals but not on the nature of the surface species observed, as indicated by the presence of bands attributed to carbonyls, formates, and carbonates. In addition, a sharp peak at 3015 cm^–1^ is observed at temperatures above 300 °C, which we assign to the C–H stretching mode of gas-phase CH_4_. Similarly, water is detected above 350 °C as indicated by the presence of a broad band in the 3700–3450 cm^–1^ range, corresponding to the symmetric and asymmetric stretching of H-bonded hydroxyl groups.^61^ The identification of such broad band can also be related to the presence of large coverages of water on the catalyst surface or the accumulation of water in the reaction chamber as a product of the CO_2_ methanation reaction. Several bands from the spectra shown in Figure 2 have been selected to monitor the evolution of carbonyls (2015 and 1920 cm^–1^), formates (2830 cm^–1^), and carbonates (1280 cm^–1^) with temperature. Note that the characteristic formate bands within 2930–2850 cm^–1^ exhibit a redshift when temperature is increased, which is attributed to the weakening of the C–H bond due to redox changes in the adsorption site. The evolution of band intensities from the selected signals with temperature is presented in Figure 3.

The generation of formates and bidentate carbonates is strongly influenced by the reaction temperature. In particular, their signals peak at 200 and 300 °C, respectively, and then drop. The carbonyl signals also increase slightly from 25 to 200 °C, and then remain constant until 400 °C. This indicates that carbonyls are not only formed on ruthenium at room temperature but also at higher temperatures, and they remain on the catalyst surface even when exposed to CO_2_ methanation temperatures (above 170 °C). The decrease of the formate, carbonate, and carbonyl bands above *ca*. 200, 300, and 400 °C, respectively, can be attributed to their hydrogenation onset or thermal decomposition. Panagiotopoulou et al.^35^ confirmed that Ru-carbonyls are reaction intermediates in the CO_2_ methanation on Ru/TiO_2_ catalysts, while other authors^30,63^ reached the same conclusion for Ru/Al_2_O_3_. However, Wang et al.^43^ concluded that formates are the main reaction intermediates. On the contrary, Upham et al.^64^ pinpointed carbonates as active intermediates of CO_2_ methanation while ruling out the participation of CO and formate-type adsorbates in a Ru-doped ceria catalyst with composition Ru_0.05_Ce_0.95_O*_x_*. This controversy highlights that particular features of the ruthenium catalyst and the experimental techniques employed to investigate the CO_2_ methanation have a major impact on the nature of the intermediates formed and, therefore, the conclusions drawn about the reaction mechanism.

### 3.3 *In Situ* NAP–XPS Experiments

**Figure 4:**
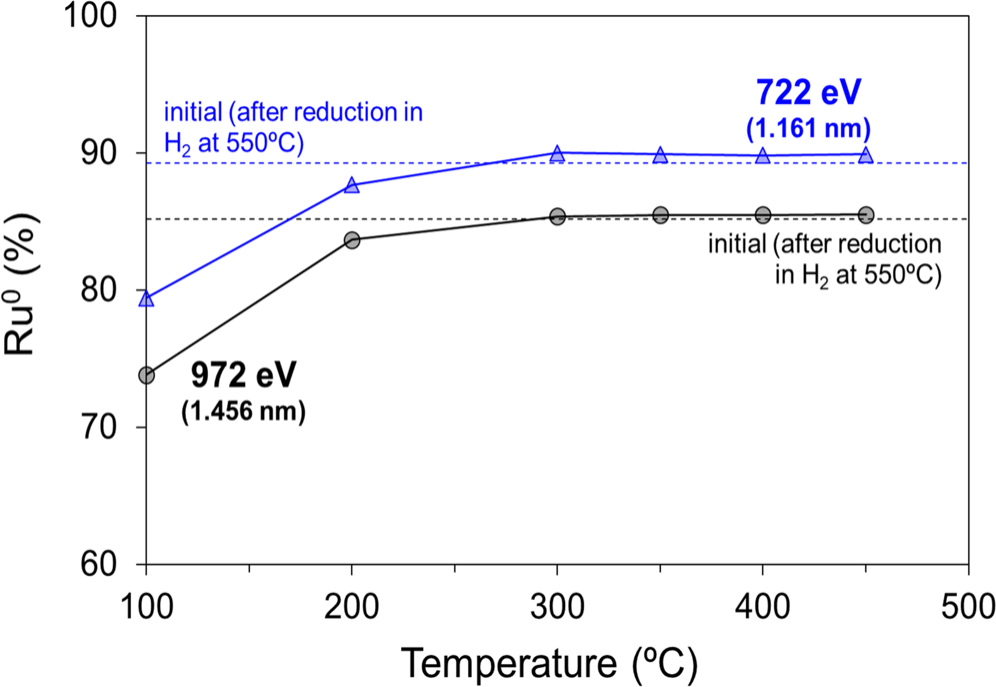
Evolution of Ru^0^ content relative to the total ruthenium as measured by NAP–XPS under CO_2_ methanation gas mixture (10% CO_2_, 40% H_2_, 50% N_2_) after a reduction pretreatment at 550 °C with 50% H_2_/N_2_. The photon energies and probed depths (estimated as 3 times the inelastic mean free path, IMFP) are also indicated. These data can be found in the Supporting Information (Table S1) for selected crystalline phases.

**Figure 5:**
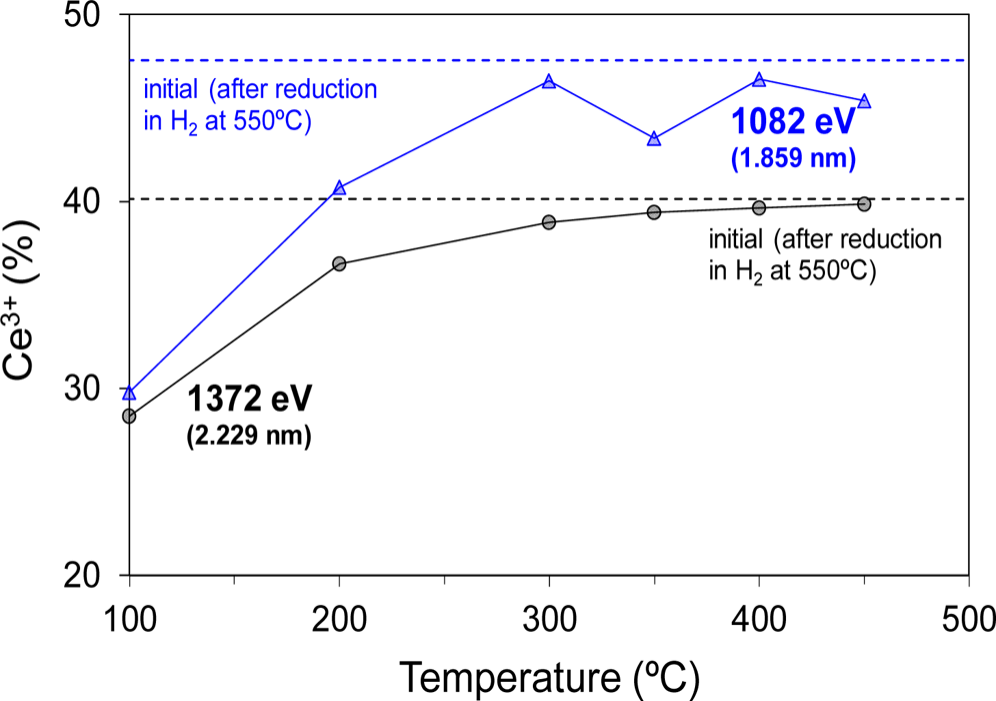
Evolution of Ce^3+^ percentage relative to the total cerium as measured by NAP–XPS under CO_2_ methanation gas mixture (10% CO_2_, 40% H_2_, 50% N_2_) after a reduction pretreatment at 550 °C with 50% H_2_/N_2_. The photon energies and corresponding sample probed depths (estimated as 3 times the IMFP) are also indicated. Data for selected crystalline phases can be found in the Supporting Information (Table S1).

The spectra recorded under steady-state conditions for Ce 3d and Ru 3d XPS regions are shown in the Supporting Information (see Figures S4 and S5, respectively). From these spectra, the percentages of Ru^0^ and Ce^3+^ were calculated,^65^ and the results are plotted as a function of temperature in Figures 4 and 5, respectively. After the reduction pretreatment, NAP–XPS reveals that the surface of the ruthenium catalyst is mainly reduced, with *ca*. 90% Ru^0^ as measured with the 722 eV photon energy (see Figure 4 and Table S2). This percentage is slightly lower (86%) when probing deeper regions with an incident energy of 972 eV. The presence of surface oxidized Ru after the reduction treatment can be related to the presence of ceria, which promotes the stabilization of oxidized species at the Ru–CeO_2_ interface.

The introduction of the methanation reaction gas mixture at 100 °C leads to the oxidation of the ruthenium surface as evidenced by the drop of the Ru^0^ percentages from 90 and 86 to 79 and 74% for the 722 and 972 eV incident energies, respectively. This observation is consistent with the dissociative chemisorption of CO_2_ leading to Ru-carbonyls and oxygen atoms, which could potentially oxidize partially the surface as observed by DRIFTS. As the temperature increases, the percentages of Ru^0^ increase alongside CO_2_ consumption up to similar values to those observed during H_2_ pretreatment (see Figure 4). Hence, we conclude that the oxygen atoms left on ruthenium upon dissociative chemisorption of CO_2_ at low temperatures (*i.e*., below 200 °C) are removed at higher temperatures in the form of H_2_O and/or transferred to ceria.

The percentage of surface Ce^3+^ cations determined by NAP–XPS experiments, shown in Figure 5, amounts to 37% after the reduction pretreatment at 550 °C under H_2_, regardless of the probed depth. This percentage decreases when the methanation gas mixture is fed into the reaction cell at 100 °C in accordance with the behavior of ruthenium, evidencing that CO_2_ chemisorption oxidizes both ruthenium and ceria. The oxidation of Ce(III) to Ce(IV) under these conditions can occur via the adsorption and dissociation of CO_2_ to CO + *O on the reduced surface of ceria, as confirmed by DFT calculations (*vide infra*). According to Figure 5, the change in concentration of Ce^3+^ (from 37% after H_2_ pretreatment to 25–14% at 100 °C in CO_2_ + H_2_) is higher than that for Ru (from 90–86 to 79–74%, respectively, as seen in Figure 4). Hence, considering that there is much more ceria than ruthenium in the Ru/CeO_2_ catalyst, we posit that the oxygen generated upon CO_2_ dissociation, which goes onto ceria, is much larger than that accumulated on ruthenium, highlighting the key role of ceria as oxygen reservoir.

As observed in Figure 5, increasing the temperature beyond 100 °C leads to the reduction of the ceria surface up to the level achieved with the H_2_ reduction pretreatment. The monitorization of the oxidation state of bulk cerium in a Ru/CeO_2_ catalyst under CO_2_ methanation conditions using synchrotron radiation was reported by Wang et al.^43^ Therein, operando XANES experiments showed that the concentration of Ce^3+^ increased from 3% at room temperature to 9% at 400 °C under reaction conditions. We note that these values are much lower than the ones obtained in this work using NAP–XPS (Figure 5), which we attribute to XANES being a bulk-sensitive analysis while XPS is surface-sensitive.^67^ Therefore, we conclude that ceria bulk remains mainly oxidized, whereas the surface is highly reduced (37% Ce^3+^) at CO_2_ methanation temperatures.

In summary, during the CO_2_ methanation reaction, the Ru/CeO_2_ catalyst is reduced on average with a surface composition of *ca*. 90–86% Ru^0^ and 37% Ce^3+^, which is similar to the catalyst state after the H_2_ reduction pretreatment at 550 °C. This indicates that, during CO_2_ methanation, the dissociative chemisorption of H_2_ is faster than that of CO_2_, leading to a larger concentration of H atoms, which most likely remain adsorbed on the ruthenium surface.

### 3.4 DFT Calculations

#### 3.4.1 Coverage Analysis and Resting State Assessment

**Figure 6:**
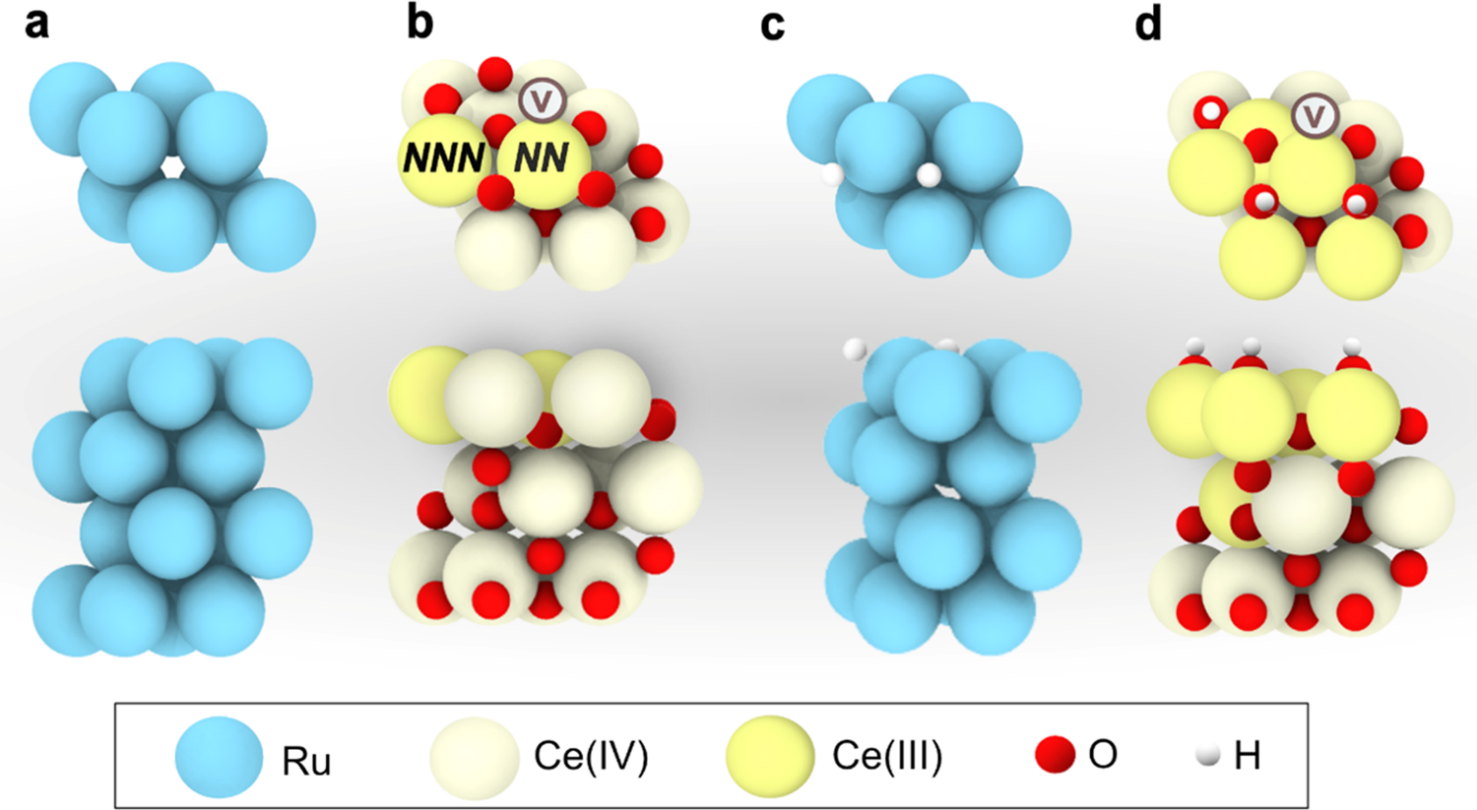
(a) Modeled Ru(0001) and (b) CeO_2–*x*_(111) surface slabs. Resting states predicted for (c) Ru(0001) and (d) CeO_2–*x*_ (111) at 225 °C. O vacancies are denoted with a circled “V”.

DFT calculations were carried out to shed light on the CO_2_ methanation pathway using representative facets of the Ru/CeO_2_ catalyst. Given the polycrystalline nature of the catalyst, as depicted by XRD (Figure S2), the lowest-energy surface slabs were selected and modeled. Namely, Ru(0001) and CeO_2_(111), which are inferred to be the most abundant and exposed terminations in the catalyst. The interaction of the surface slabs with the CO_2_ + H_2_ mixture at representative conditions was studied to assess the participation of each phase in the multistepped reaction mechanism. Because as-prepared ceria is known to exhibit oxygen vacancies, a nonstoichiometric *p*(2 × 2)-CeO_2–*x*_(111) surface with one surface oxygen vacancy was modeled, which corresponds to a 25% surface reduction. The lowest-energy configuration for this slab corresponds to two surface Ce^3+^ ions located in the nearest neighbor (NN) and next-nearest neighbor (NNN) positions relative to the oxygen vacancy, in agreement with previous theoretical studies.^67,68^ The modeled Ru(0001) and CeO_2–*x*_(111) surface slabs are depicted in Figure 6a,b, respectively.

To assess the catalyst resting state at the onset of the CO_2_ methanation reaction, the surface coverage of the CeO_2–*x*_(111) and Ru(0001) slabs was investigated by computing the Gibbs adsorption energies of all of the reactant species under relevant experimental conditions (see the Materials and Methods section for details). DFT calculations revealed that the dissociative chemisorption of H_2_ on Ru(0001) has a negligible activation barrier (see Figure S6), rendering this process extremely fast. Hence, we focused our coverage analysis of this surface on the computation of the Gibbs energy of consecutive H adsorptions as a function of temperature. The results derived from this analysis led to the prediction of a surface with all of the fcc sites occupied by H atoms as the catalyst resting state at room temperature (Figure S7). In addition, calculations revealed that, as temperature increases, lower H coverages become more stable until the clean surface prevails at temperatures above *ca*. 300 °C. At 225 °C, a temperature slightly above the experimental onset found in methanation catalytic tests (*ca*. 175 °C), the Ru surface is predicted to be partially covered with 2H atoms in fcc positions, as shown in Figure 6a. Hence, this surface termination was selected for the mechanistic studies of the CO_2_ methanation reaction on Ru(0001).

Similarly to Ru(0001), H_2_ activation on the CeO_2_(111) surface has been shown to demand a relatively low energy barrier (*ca*. 1 eV), leading to the hydroxylation of the surface in a highly exergonic process.^57^ Consequently, we modeled the CeO_2–*x*_(111) surface with different levels of hydroxylation as a function of reaction temperature (Figure S7b), finding a coverage with 3OH groups and 1O vacancy (see Figure 6b) as the most likely surface termination within the experimental temperature range of 25–360 °C. Hence, we selected this surface coverage to investigate the CO_2_ methanation mechanism on CeO_2–*x*_(111).

#### 3.4.2 CO_2_ Methanation on Ru(0001)

The binding of a CO_2_ molecule was investigated on all possible sites of the Ru(0001) surface. The most favorable adsorption sites are depicted in Figure S8. On the clean Ru(0001) surface, we found that CO_2_ is weakly adsorbed in a V-shape on an fcc site with a Gibbs energy of +0.15 eV, while CO_2_ adsorption is hampered on the H-covered Ru(0001) surfaces. In particular, CO_2_ remains physisorbed at 4.02 Å on the 4H-fcc surface as there are no fcc sites available in this coverage. Hence, a H coverage blocks CO_2_ adsorption and only when fcc sites become available at high temperatures, CO_2_ can be adsorbed. This remark points out that CO_2_ adsorption and activation on Ru can be limited at the initial CO_2_ methanation conditions and conditioned by the H coverage.

**Figure 7:**
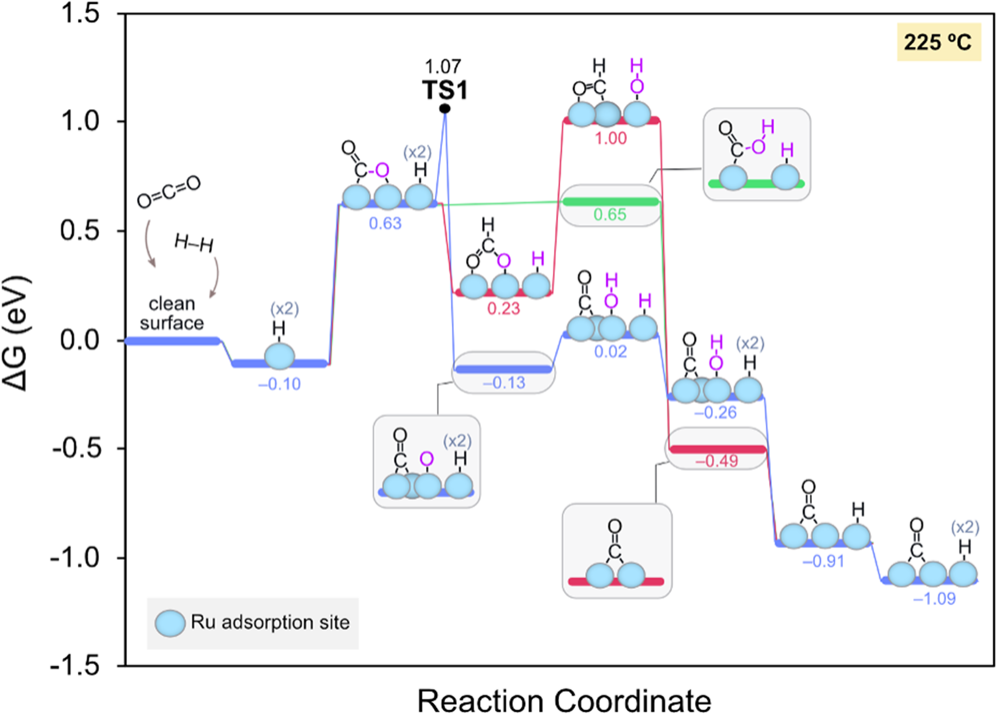
Calculated Gibbs energy diagram for the formation of carbonyl groups on Ru(0001) under CO_2_ methanation conditions at 225 °C.

After determining the 2H-covered Ru(0001) surface as the catalyst resting state under relevant CO_2_ methanation conditions, the potential CO_2_ evolution pathways were investigated at 225 °C. The lowest Gibbs energy diagrams obtained for the formation of Ru-carbonyls observed in experiments are depicted in Figure 7. These pathways involve the hydrogenation of CO_2_ by the surface H atoms followed by the restitution of the H coverage as the dissociative chemisorption of H_2_ has been shown to be extremely fast and highly exergonic.

As shown in Figure 7, after CO_2_ adsorption on the H-covered surface, CO_2_ can either split into *CO and *O or be hydrogenated to other species, namely, formate (*OCHO*, red trace) or hydrocarboxylate (*COOH, green trace). Ru(0001) surfaces displaying high H coverages have been reported to favor CO_2_ hydrogenation over CO_2_ dissociation since the former frees one active site, whereas the latter requires an additional surface site for the binding of the generated *O.^66^ However, because a partial H coverage is predicted under our experimental reaction conditions, we find that CO_2_ dissociation is the most thermodynamically favored route by 0.30 eV. This step is also independent of the reaction temperature, as illustrated in Figure S9. DFT calculations indicate that the cleavage of CO_2_ is exergonic by −0.70 eV and requires a low relative barrier of 0.44 eV (see Figure S10), rendering this process feasible under experimental conditions. Importantly, we note that this step results in the local reoxidation of Ru due to the adsorption of *O, which is in line with the surface reoxidation observed by NAP–XPS. The presence of carbonyl groups on cationic Ru sites observed in the DRIFT spectra could be explained by means of this mechanism, too. However, although our theoretical findings show that CO_2_ split on Ru surfaces is thermodynamically feasible under methanation conditions, it is not accurate to attribute such cationic species to this process since the role of the support in the redox state of the Ru particles cannot be ruled out. After CO_2_ dissociation, the *O species is hydrogenated in two consecutive steps to form an *OH group and then H_2_O, which desorbs from the surface reducing Ru back to its original state.

Notably, this mechanism is considerably lower in energy than the alternative paths involving formates and their evolution via hydrocarboxylates (*COOH) or formyl groups (*CHO), which can also lead to very stable *CO groups by releasing water to the gas phase, as shown in Figure 7. This is also in line with the broad and intense Ru^*n*+^–CO band detected in *in situ* DRIFTS experiments all along the reaction course. Overall, Figure 7 shows that Ru-carbonyls are very stable and that these can be formed via different pathways, although the CO_2_ dissociative route is predicted to dominate. This may explain the broad and irregular shape of the carbonyl band in the DRIFT spectra, indicating the accumulation of CO in different local environments on the surface.

**Figure 8:**
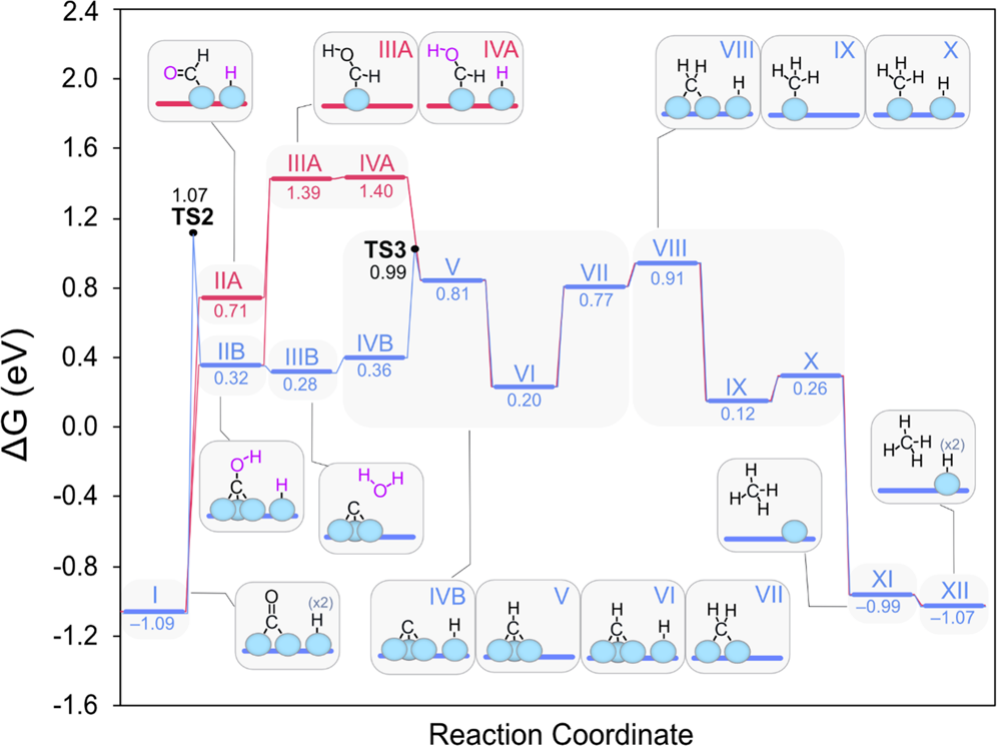
Calculated Gibbs energies for different CO evolution pathways on Ru(0001) under methanation conditions at 225 °C.

Once the stable *CO species has been formed on the Ru(0001) surface, these can be hydrogenated to *COH or *CHO, which have been proposed to be further hydrogenated to CH_4_ in a rate-determining process.^66^ As can be observed in Figure 8, our calculations indicate that CO hydrogenation to *COH is more favorable by *ca*. 0.40 eV and demands an energy barrier of 2.16 eV (see Figure S11 for the TS2 calculation), which is considerably higher than that for CO_2_ dissociation. This observation could explain the carbonyl formation at room temperature and their constant concentration on surface up to 400 °C. Subsequently, *COH undergoes hydrogenation to *C and H_2_O, which desorbs into the gas phase in an almost thermoneutral process. From this point, the adsorbed *C evolves as the final product CH_4_ through consecutive hydrogenations involving exergonic or moderately endergonic steps. One of the endergonic steps of the mechanism corresponds to the hydrogenation of *C to *CH, although this process exhibits a relative energy barrier of only 0.63 eV (difference between TS3 and IVB). Therefore, we assume low comparable barriers for the successive stepwise hydrogenations.

Overall, because of the marked stability of the Ru-carbonyls, we conclude that *CO to CH_4_ hydrogenation requires a global activation energy of *ca*. 2.0 eV, which corresponds to the formation of *COH via TS2. This high energy barrier is in good agreement with *in situ* experimental DRIFTS, which shows that the Ru–CO band is already intense at room temperature and remains unaltered throughout the reaction course. In addition, there is no sign of CO_2_ conversion at temperatures below the CO_2_ methanation onset and no CO is detected in the outlet streams, pointing to *CO hydrogenation as the RDS. Hence, both experiments and theoretical studies indicate that CO is strongly bound on Ru(0001), where it accumulates until the temperature is high enough (CO_2_ methanation onset is at *ca*. 175 °C) to hydrogenate *CO to CH_4_. The effect of temperature is observed in Figure S9, where the most favored mechanistic route remains the same at 425 °C. In conclusion, *CO adsorbs on the H-covered Ru(0001), the first hydrogenation takes place, and the formation of subsequent intermediates is thermodynamically driven.

#### 3.4.3 CO_2_ Methanation on CeO_2–*x*_(111)

After modeling the CO_2_ methanation mechanism on Ru(0001), we turned our attention to ceria using the resting state predicted above with 3OH groups and 1O vacancy.

**Figure 9:**
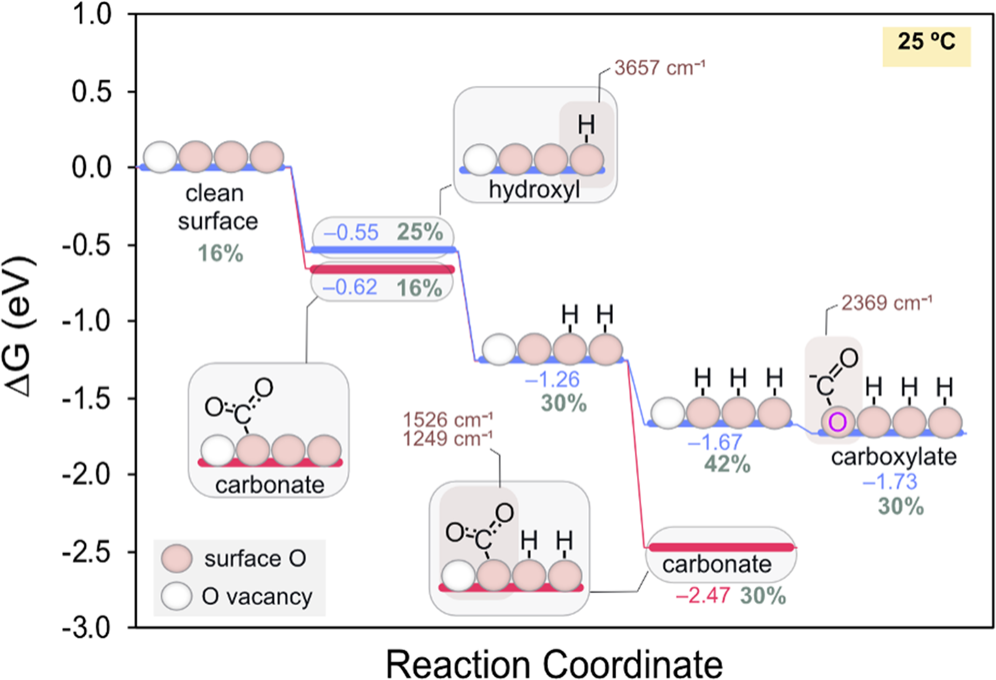
Calculated Gibbs energies at 25 °C for the most favorable adsorption modes of CO_2_ on the CeO_2–*x*_(111) surface with 1O vacancy under the predicted H coverage. Computed vibrational frequencies are shown in brown, while the % Ce^3+^ in the CeO_2–*x*_(111) surfaces is displayed in gray.

**Figure 10:**
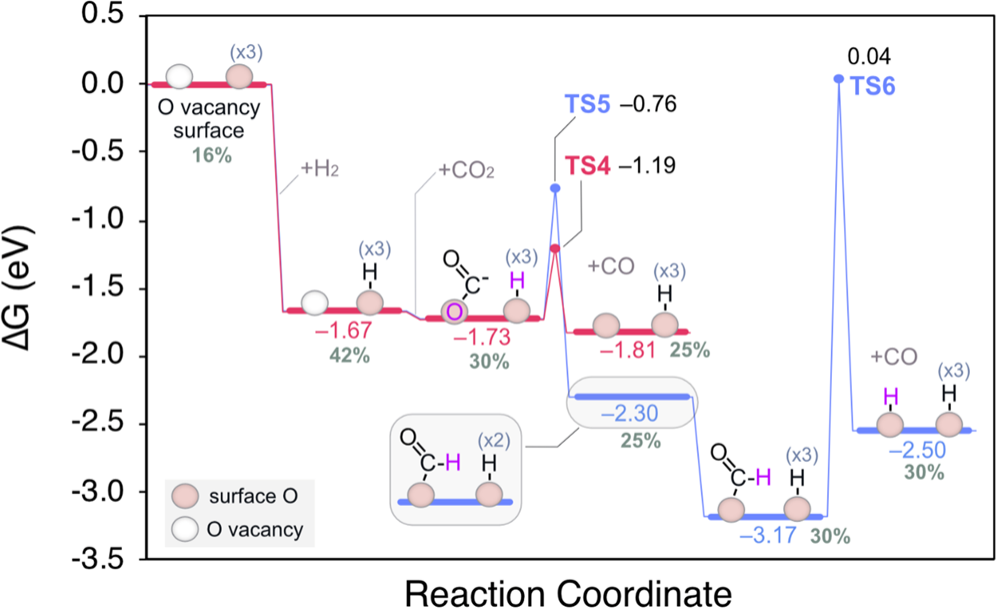
Calculated Gibbs energy diagram for the formation of carboxylates and their decomposition pathways at 25 °C. The % Ce^3+^ on the slab at each reaction step is shown in gray.

In contrast to Ru, CeO_2_ has good affinity for CO_2_ retention,^69,70^ which further grows with the presence of reduced Ce^3+^ cations. As shown in Figure 9, the binding of CO_2_ onto a surface oxygen to form a carbonate species (*OCOO) is exergonic by −0.62 eV on the reduced surface. However, we note that, in the presence of H_2_, these two processes can compete since both hydroxylation and carbonate formation require an available surface oxygen. Interestingly, unlike with Ru(0001), the presence of surface OH groups favors the formation of carbonates. In fact, the 2*OH + *OCOO state is more stable than the coverage with only 3*OH (−2.47 vs −1.67 eV). Therefore, *OCOO and *OH groups are expected to coexist on ceria, in good agreement with DRIFTS. Alternatively, CO_2_ can also bind on the oxygen vacancy site in the 3*OH surface as a V-shaped carboxylate species with an energy of −1.73 eV. This adsorption mode is especially relevant since the oxygen vacancy is partially refilled by one of the oxygen atoms from CO_2_, leading to the reoxidation of ceria as observed by NAP–XPS. These carboxylates may evolve in two different pathways as shown in the Gibbs energy diagram presented in Figure 10, namely, via direct splitting into CO and O, which directly refills the oxygen vacancy (red trace), or through the hydrogenation to formates (blue trace).

We note that the resulting CO released via the carboxylate route is not chemisorbed on ceria according to DFT calculations, neither released to the gas phase since no CO was detected in the outlet gases. Therefore, it is very likely that the weakly physisorbed CO interacts with the Ru phase at the triple-phase boundary of the Ru/CeO_2_ catalyst giving rise to Ru-carbonyls. Nonetheless, given the low energy barrier (TS4) found for the splitting of carboxylates on ceria (0.54 eV; see Figure S13), we conclude that carboxylates are a potential additional source of Ru-carbonyls at room temperature.

Our calculations also predict that carboxylates can be largely stabilized as formates with a slightly larger activation energy of 0.97 eV via TS5 (see Figure S14) and remain on surface until sufficient energy is provided for their decomposition into CO (Figure 10, blue trace). Importantly, while formates are thermodynamically much more stable than carboxylates, they are predicted to be rather kinetically inert, in agreement with the observation of the depletion of the formates band in DRIFTs above 200 °C. Finally, in contrast to formates, carbonates may be merely released as CO_2_ as the temperature increases, whose depletion is triggered beyond 300 °C according to DRIFTS.

Overall, theoretical calculations reveal that ceria can source CO to the ruthenium catalyst at low and high temperatures, giving rise to Ru-carbonyls in addition to the intrinsic capacity of Ru to split CO_2_ with a moderate activation energy. This highlights the important role of ceria as an oxygen sink by assisting in the Ru-catalyzed reaction mechanism, which is not possible with inert supports like Al_2_O_3_ or SiO_2_ rendering a poorer catalytic performance. Besides, these results shed light on why Ru/CeO_2_ catalysts with Ru and CeO_2_ phases in tight interaction exhibit improved catalytic performance for the CO_2_ methanation reaction, in good agreement with experiments. Simulations show that Ru on its own can promote both H_2_ dissociation and CO_2_ methanation, while the ceria support facilitates CO_2_ dissociation. Since the binding of CO_2_ on Ru metal is weak and even more difficult on the H-covered surface, the assistance of ceria is very beneficial to accelerate the CO_2_ methanation rate at temperatures around the reaction onset. Future modeling studies of the Ru–Ce interfacial sites should confirm the key role of synergistic interactions arising in Ru-supported cerium oxide catalysts.

## 4 Conclusions

This work has investigated the catalytic behavior of a high-performance Ru/CeO_2_ catalyst on the CO_2_ methanation reaction by means of *in situ* spectroscopic techniques and periodic DFT calculations of the individual Ru and CeO_2_ phases under representative reaction conditions. Exposure of the CO_2_ + H_2_ methanation mixture on the Ru metal and CeO_2_ results in the partial reoxidation of these surfaces via CO_2_ dissociative chemisorption at room temperature, as confirmed by NAP–XPS. The CO_2_ chemisorption on both Ru and CeO_2_ modeled by DFT supports the oxidative nature of the process.

H_2_ activation and dissociation under CO_2_ methanation conditions can occur on both the Ru phase, in an almost barrierless process, and on CeO_2–*x*_(111), with a low activation energy, leading to high H and OH coverages, respectively. For ceria, surface hydroxylation is beneficial for CO_2_ retention, while the opposite is predicted for Ru. This complementarity seems to have a positive influence on the overall reactivity, enabling additional CO_2_ adsorption sites which are predicted to be accessible to Ru at the Ru/CeO_2_ interface. The overall rate-determining step in the CO_2_ methanation mechanism on the Ru/CeO_2_ catalyst is the hydrogenation of Ru-carbonyls with an energy barrier of 2.16 eV.

On ceria, we have shown that CO_2_ is accumulated on the surface in the form of strongly bound carbonates, while carboxylates sitting on oxygen vacancies are thermodynamically driven to the generation of formate species. The evolution of the latter stable species requires a considerable activation energy, and hence, they remain adsorbed on the ceria surface up to 200 °C. Beyond this temperature, formates can decompose to CO and yield ruthenium carbonyls, as observed by DRIFTS. On the other hand, at lower temperatures, carboxylates are kinetically very reactive leading to CO and O*, restoring the oxygen vacancy and giving rise to the Ru-carbonyls observed at room temperature via a spillover mechanism.

Although Ru shows a very weak CO_2_ binding unlike CeO_2_, an incipient Ru reoxidation is also observed in CO_2_ methanation reaction conditions even at room temperature. This Ru oxidation must be related to the synergistic Ru–CeO_2_ interactions, either by O spillover from ceria at the interfacial sites or by enabling highly active interfacial sites, which split CO_2_ and stabilize CO very strongly.

## Supporting Information Available

Physicochemical characterization of the catalyst, namely, N_2_ adsorption–desorption isotherms (Table S1, Figure S1), X-ray diffraction (Figure S2) and temperature-programmed reduction with H_2_ (Figure S3, Table S2), and Ru 3d and Ce 3d XPS fitted spectra from *in situ* NAP–XPS experiments at different energies (Figures S4 and S5); and DFT calculations to support the experimental and theoretical findings (Figures S6–S15) (PDF). All the modeled structures and relevant DFT data is openly available at the ioChem-BD repository: https://doi.org/10.19061/iochem-bd-6-87
