# Supplementary material for: Elucidating the Role of the Metal Catalyst and Oxide Support in the Ru/CeO2-Catalyzed CO2 Methanation Mechanism
Source: J Phys Chem C Nanomater Interfaces. 2021 Nov 16;125(46):25533–44. doi: 10.1021/acs.jpcc.1c07537 (PMC8631708; doi:10.1021/acs.jpcc.1c07537)
Supplement: Supplementary file 1 — jp1c07537_si_001.pdf [file jp1c07537_si_001.pdf]

Supporting Information

Elucidating the Role of the Metal Catalyst and Oxide Support in the Ru/CeO_2 Catalyzed CO_2 Methanation Mechanism

Sergio López-Rodríguez[a], Arantxa Davó-Quiñonero[a,b*], Esther Bailón-García[a], Dolores Lozano-Castelló[a], Facundo C. Herrera[c,d], Eric Pellegrin[c,†], Carlos Escudero[c], Max García-Melchor[b,*] and Agustín Bueno-López[a*]

[a]*Inorganic Chemistry Department, University of Alicante, Carretera San Vicente del Raspeig s/n E-03080, Alicante, Spain.*

[b]*School of Chemistry, CRANN and AMBER Research Centres, Trinity College Dublin, College Green, Dublin 2, Dublin, Ireland;*

[c]*ALBA Synchrotron Light Source, Carrer de la Llum 2-26, 08290 Cerdanyola del Vallès, Barcelona, Spain*

[d]*Instituto de Investigaciones Fisicoquímicas Teóricas y Aplicadas (INIFTA, CONICET), Departamento de Química, Facultad de Ciencias Exactas, Universidad Nacional de La Plata, Diagonal 113 y 64, 1900 La Plata, Argentina.*

[†]*Current address: Carl Zeiss SMT GmbH, Rudolf-Eber-Straße. 2, 73447 Ober-kochen, Germany.*

Contents

Catalyst characterization S2

In situ NAP–XPS experiments S7

DFT calculations S12

References S20

Catalyst Characterization

N_2 adsorption-desorption isotherm was obtained at –196 ºC (Autosorb-6, Quantachrome) after outgassing the catalyst under vacuum at 200 ºC for 4 hours (Figure S1). The metal content was measured by inductively coupled plasma-optical emission spectroscopy (ICP-OES) in a PerkinElmer device (Optima model 4300 DV) after digestion in a HCl/HNO3 (3:1 volume) mixture assisted by microwaves.

The crystalline structure was analyzed by X-ray diffraction in a Rigaku Miniflex II diffractometer. The diffractograms were recorded in a range of 2θ from 10º to 90º, with a step size of 0.025º. The wavelength used was λ = 0.155418 nm corresponding to the CuK-alfa radiation. The average crystal size of ceria was determined using the Scherrer equation.

The reducibility of the catalyst was examined by H_2-temperature programmed reduction (H_2-TPR) in a Micromeritics Pulse ChemiSorb 2705 device. 20 mg of catalyst were placed in a tubular quartz reactor coupled to a TCD detector and 35 mL/min of 5% H_2 were fed at a heating rate of 10 ºC/min.

Table S1 shows catalyst characterization results obtained from ICP, XRD, N_2 adsorption and reaction tests.

Table S1. Structural and reaction parameters the as-synthesized materials.

Sample	Ru content (w. %)[a]	Cell parameter (nm)[b]	Crystallite Size (nm) [b]	B.E.T (m^2g^{-1}) [c]	Reaction rate ($mol.g_{cat}^{-1}s^{-1}$) [d]
CeO_2	–	0.5423	14	50	–
Ru/CeO_2	4.1	0.5400	13	68	$2.22\cdot10^{-5}$

[a] ICP
[b] XRD
[c] N_2 physisorption
[d] measured at 170 ºC

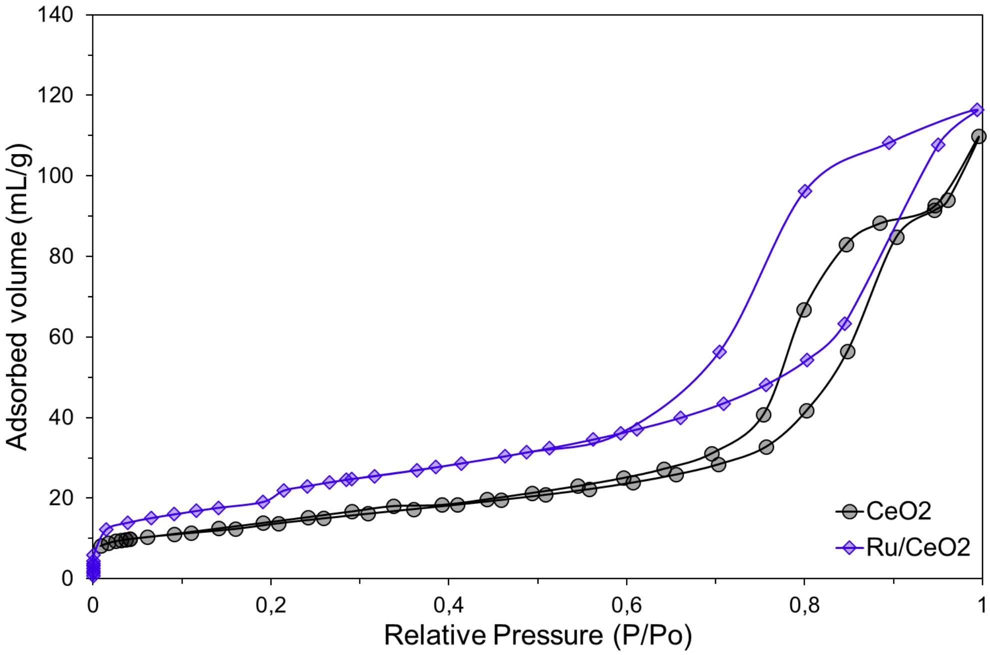

Figure S1. N_2 physisorption isotherm at –196 ºC of fresh catalysts after outgassing at 200 ºC for 4 hours.

The specific surface area of the catalyst was examined by N_2 adsorption-desorption at –196 ºC. Figure S1 shows the isotherms of the Ru/CeO_2 catalyst and bare ceria, included for comparative reasons. Both display micro-mesoporosity with BET areas in the range from 50–70 m^2/g.[1–3] On the one hand, ceria support displays a H2(b) hysteresis loop caused by capillary condensation phenomena that evidences broad pore size distribution, as expected upon the nitrate decomposition method. On the other hand, the lack of a plateau in the last points of adsorption branch also proves the presence of macroporosity. Comparing between CeO_2 and Ru/CeO_2 samples, the presence of Ru induces a slight increase in the calculated BET surface from bare CeO_2, probably due to partial pore opening upon Ru loading process.

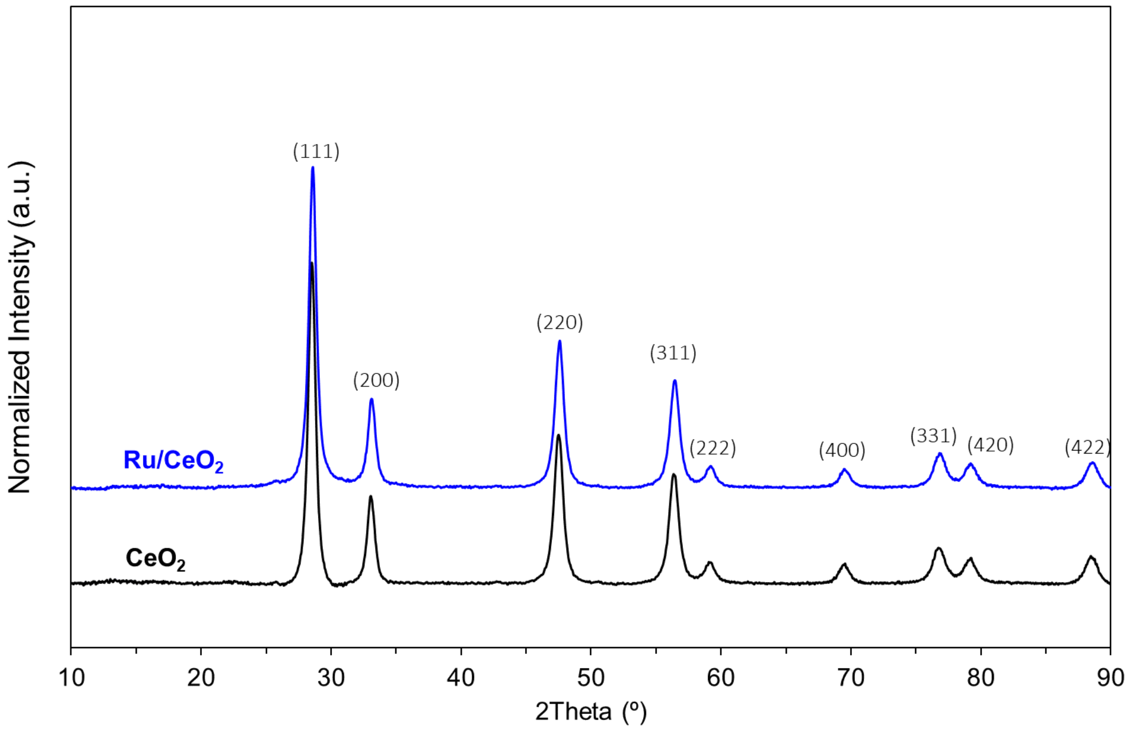

Figure S2. X-ray diffractogram of the as-prepared materials.

The crystalline structure was determined by X-Ray Diffraction and the results of the main parameter such as lattice parameter and crystallite size are compiled in the Table S1. According to Figure S2, for both samples the peaks observed are aligned with the fluorite structure of ceria with the dominant CeO_2 (111) crystal facets. The peaks located at 28.5º, 33.1º, 47.6º are indexed at JCPDS file #34-0394. The absence of any ruthenium/ruthenium oxide peak can be ascribed to the well-dispersed particles on the surface of ceria.

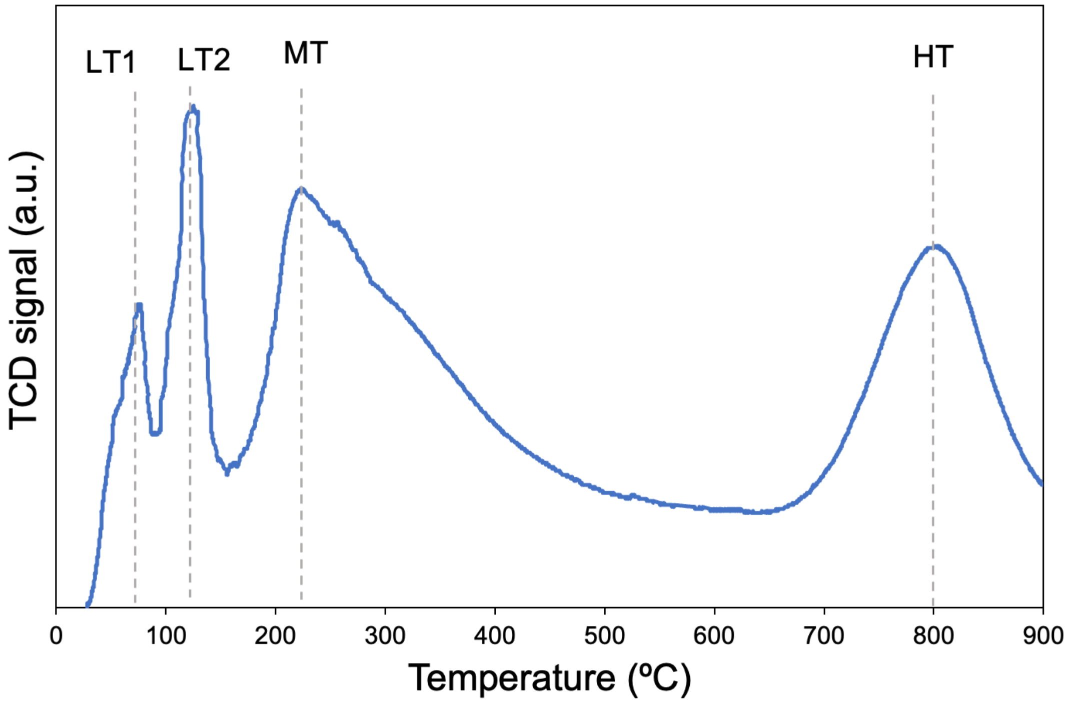

Figure S3. Reduction profile of the Ru/CeO_2 catalyst in the H_2-TPR experiment.

Noticeably, the reduction of surface Ce ions shifts from *ca.* 500 ºC, as reported, to the low 220 ºC, while bulk reduction occurs at *ca.* 800 ºC. The two low temperature (LT) peaks at 75 and 125 ºC are attributed to the reduction of finely dispersed and bigger bulk-like Ru particles, respectively. However, a heterogeneous range of Ru cationic species with different interactions with the support cannot be ruled out as inferred from the shoulders in the LT peaks shape. According to Table S2, all the Ru^{4+} cations are reduced to Ru metal in a mol ratio H_2/Ru higher than 2, indicating that part of the surface support cations is also being reduced. At higher temperatures, the consumption of hydrogen is only attributed to the Ce^{4+} cations. The MT peak with centre at *ca*. 220 ºC is ascribed to the ceria surface reduction, which is typically observed around 500 ºC for metal-free ceria materials. Finally, the reduction peak at *ca*. 800 ºC is attributed to the reduction of Ce^{4+} cations in the bulk.

Table S2. H_2 uptake calculated as mmol H_2/mmol Ru (*) and mmol mmol H_2/mmol CeO_2 (†) from the reduction peaks of Ru/CeO_2 catalyst.

Sample	LT peaks (< 200 ºC)	MT peak (200–600 ºC)	HT (> 600 ºC)
Ru/CeO_2	2.03(*)	0.12 (†)	0.08 (†)

In Situ NAP–XPS Experiments

NAP–XPS experiments were performed using different photon energies in order to obtain information from different surface depths. Table S2 compiles the inelastic mean free paths of pure solid phases corresponding to each energy utilised.

Table S3. Inelastic electron mean free paths (IMFP) for pure solid phases of Ru and Ce corresponding to each of the photon energies used in NAP-XPS experiments.[2]

Phase	Photon energy (eV)	IMFP (Å)
Ru	972	14.1
Ru	722	11.4
RuO_2	972	16.9
RuO_2	722	13.6
Ce_2O_3	1372	22.3
Ce_2O_3	1082	18.6
CeO_2	1372	21.8
CeO_2	1082	18.2

Figures S4a and S4b show the deconvoluted Ce 3d XPS region spectra recorded under different conditions and the fitting assignment based on the standard nomenclature provided by Burroughs et al.[3] The Ce^{3+} percentage was calculated as the ratio of the sum of the intensities of the u_0, u', v_0, and v' peaks with respect to the sum of the intensities of all the contributions from the Ce 3d region. Figures S5a and S5b show the Ru 3d energy region. The Ru^0 percentage was estimated as the ratio of Ru^0 with respect to the contribution of all the species (Ru^{2+} + Ru^0) within the Ru 3d region.

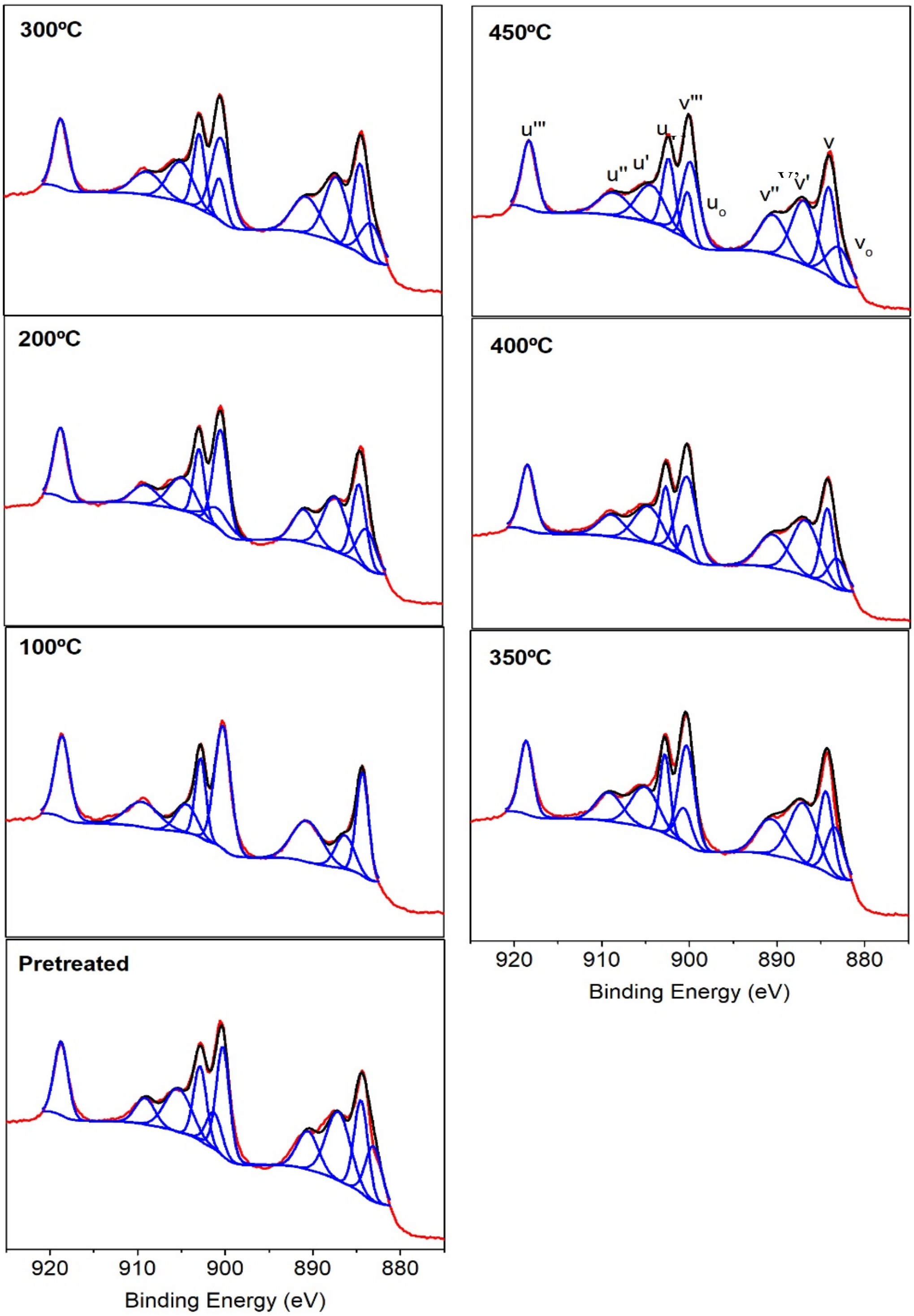

Figure S4a. In situ NAP–XPS measurements, performed with 1372 eV photon energy, showing the evolution at increasing temperatures of the Ce 3d region from 100 ºC up to 450 ºC. The catalyst was exposed sequentially to a pretreatment under 50% H_2/N_2 for 1 h at 550 ºC (pretreated spectrum). CO_2 methanation spectra recorded under of 30 mL/min (16 % CO_2, 64 % H_2, 20 % N_2), 1 mbar total pressure.

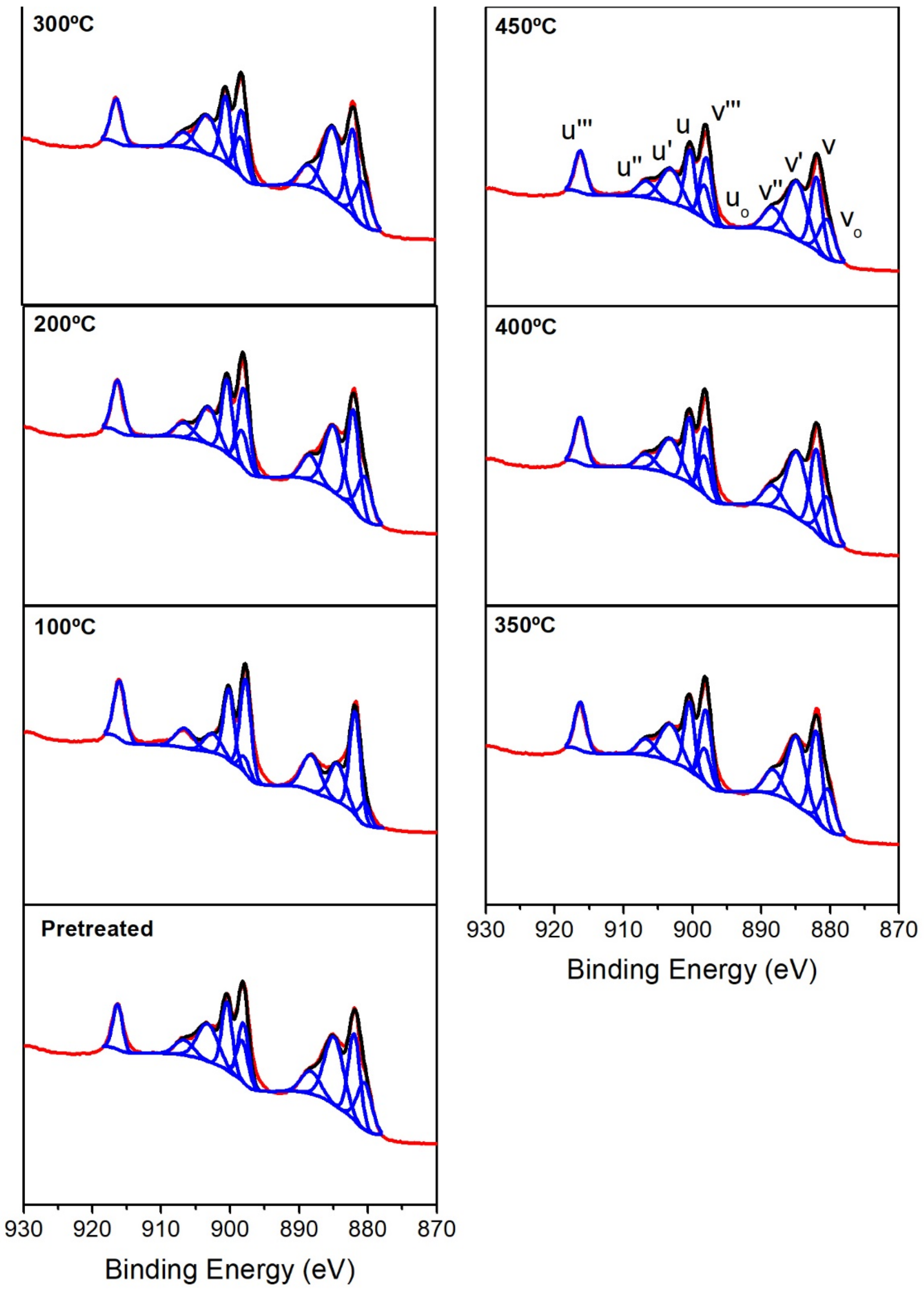

Figure S4b. In situ NAP–XPS measurements, performed with 1082 eV photon energy, showing the evolution at increasing temperatures of the Ce 3d region from 100 ºC up to 450 ºC. The catalyst was exposed sequentially to a pretreatment under 50% H_2/N_2 for 1 h at 550 ºC (pretreated spectrum). CO_2 methanation spectra recorded under of 30 mL/min (16 % CO_2, 64 % H_2, 20 % N_2), 1 mbar total pressure.

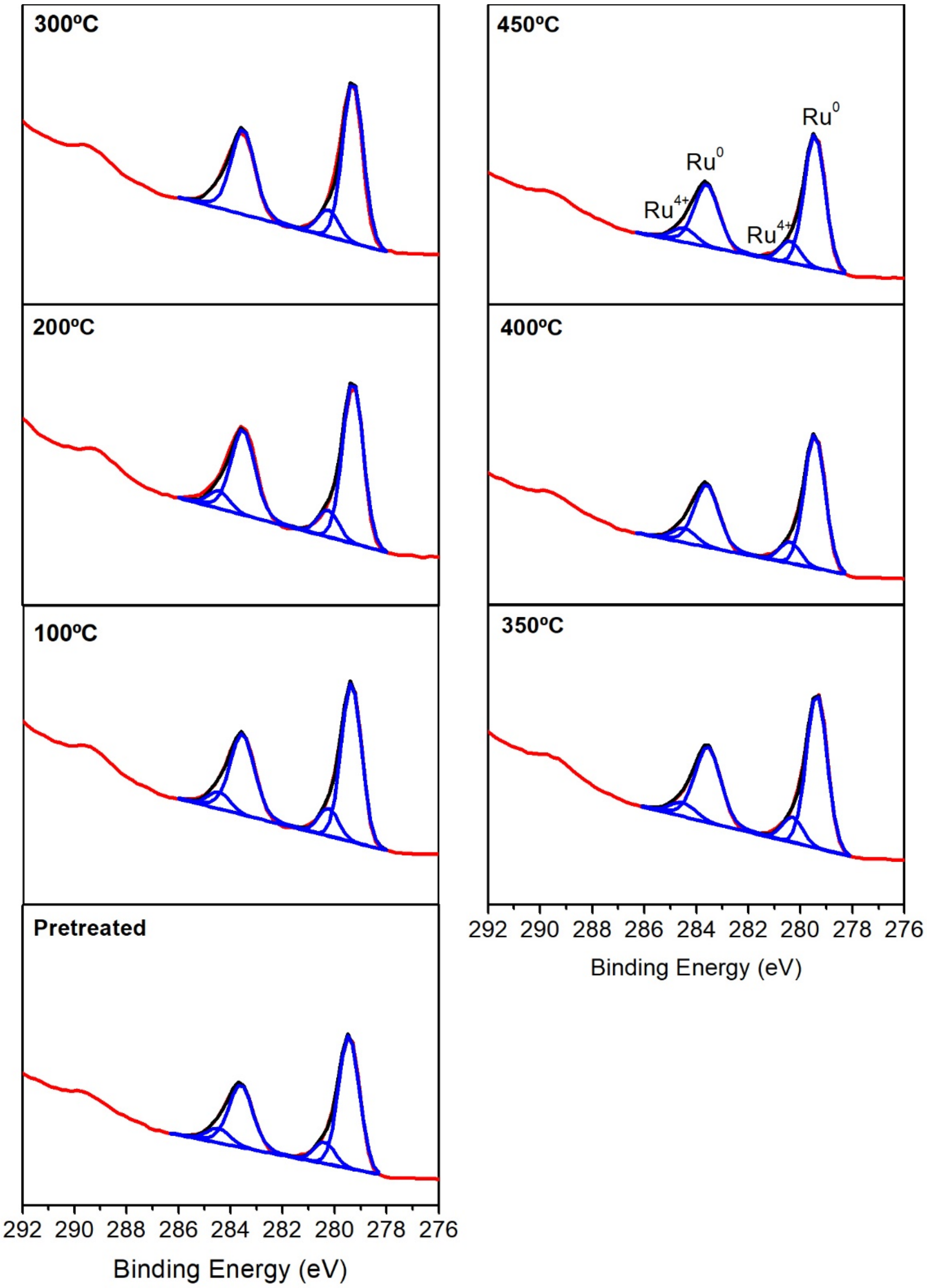

Figure 5Sa. In situ NAP–XPS measurements, performed with 972 eV photon energy, showing the evolution at increasing temperatures of the Ru 3d region from 100 ºC up to 450 ºC. The catalyst was exposed sequentially to a pretreatment under 50% H_2/N_2 for 1 h at 550 ºC (pretreated spectrum). CO_2 methanation spectra recorded under of 30 mL/min (16 % CO_2, 64 % H_2, 20 % N_2), 1 mbar total pressure.

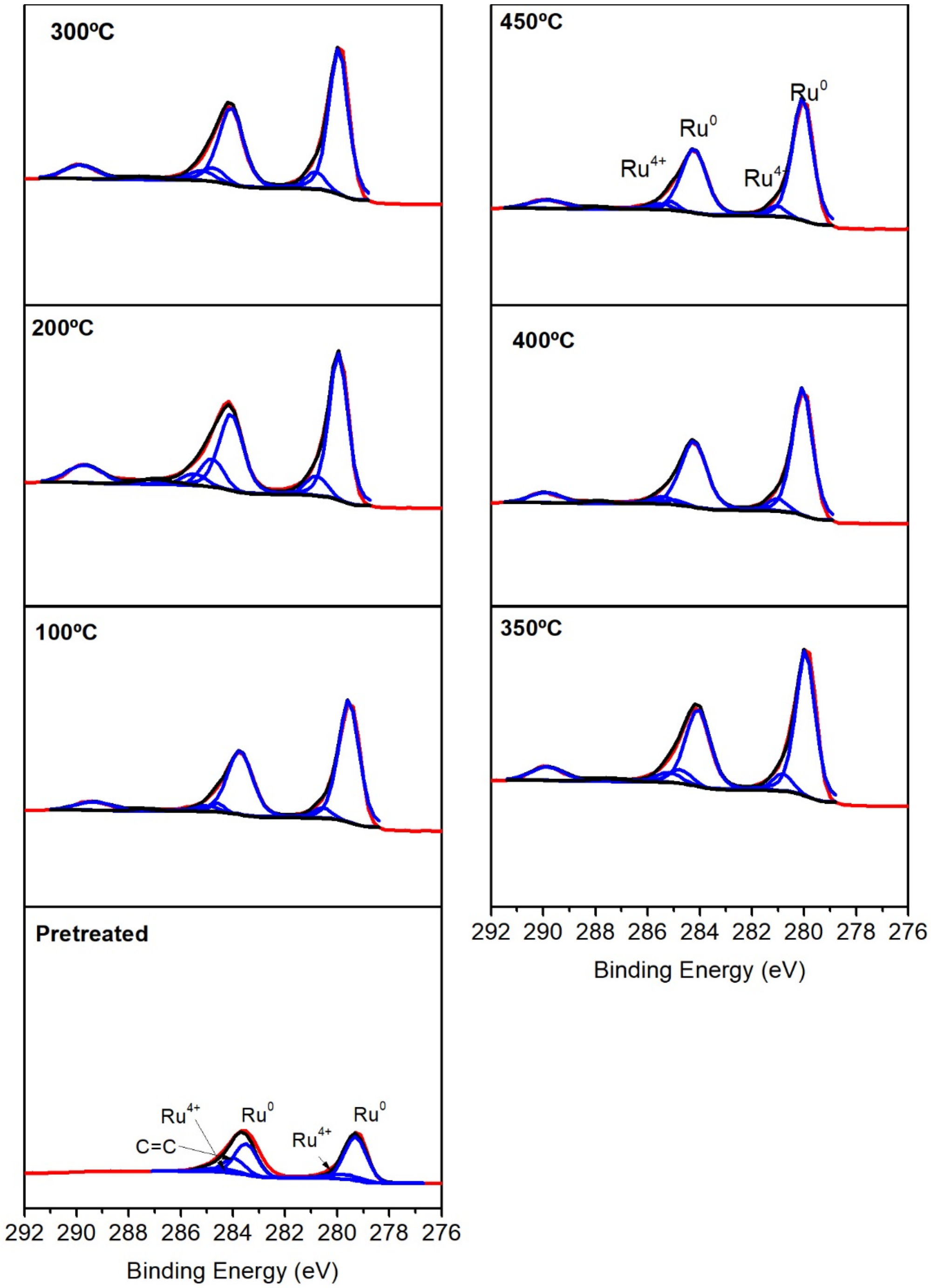

Figure S5b. In situ NAP–XPS measurements, performed with 722 eV photon energy, showing the evolution at increasing temperatures of the Ru 3d region from 100 ºC up to 450 ºC. The catalyst was exposed sequentially to a pretreatment under 50% H_2/N_2 for 1 h at 550 ºC (pretreated spectrum). CO_2 methanation spectra recorded under of 30 mL/min (16 % CO_2, 64 % H_2, 20 % N_2), 1 mbar total pressure.

DFT Calculations

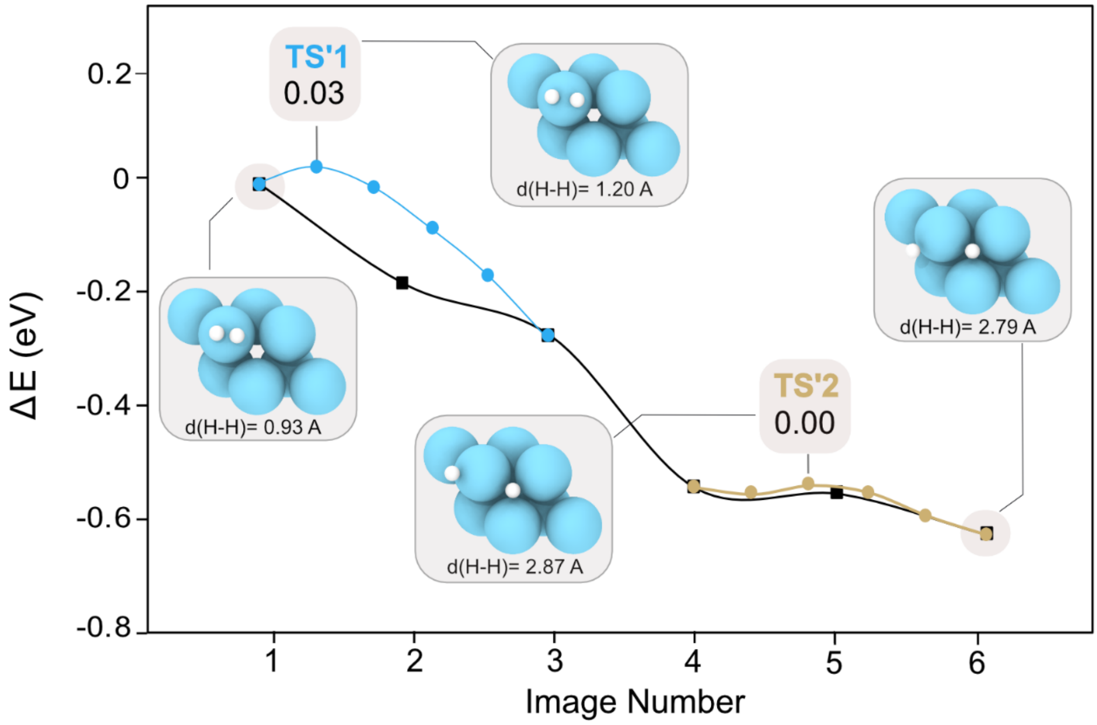

Figure S6. CI-NEB analysis of H_2 activation on Ru(0001) surface. Energy diagram referred to image 1, which corresponds to H_2 adsorption on Ru top.

As Figure S6 reveals, H_2 activation proceeds via two steps: (1) H_2 dissociation, with an activation energy of +0.034 eV (images 1-4); and (2) H migration to the *fcc* sites (images 4-6) with an activation energy of 4 meV.

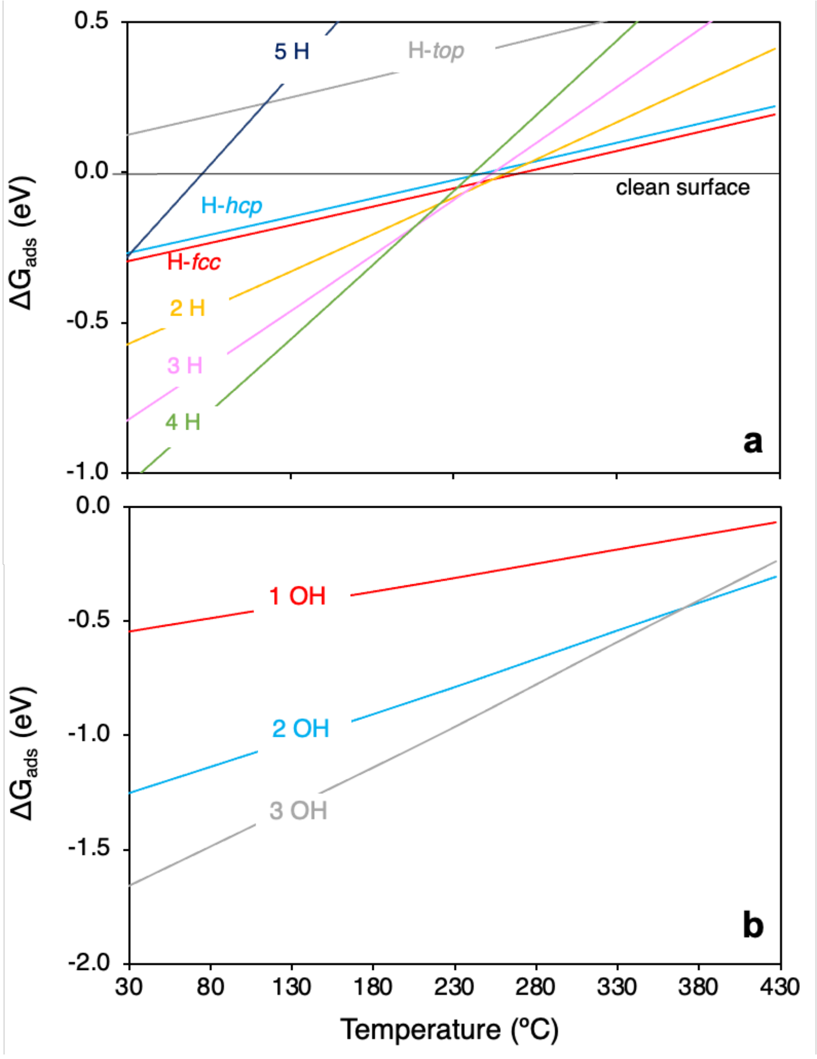

Figure S7. Gibbs energies of different surface coverages computed as a function of temperature relative to the clean slab at 0.64 bar of H_2 partial pressure for the (a) Ru(0001) surface and (b) the CeO_{2-x}(111) surface with 1 oxygen vacancy.

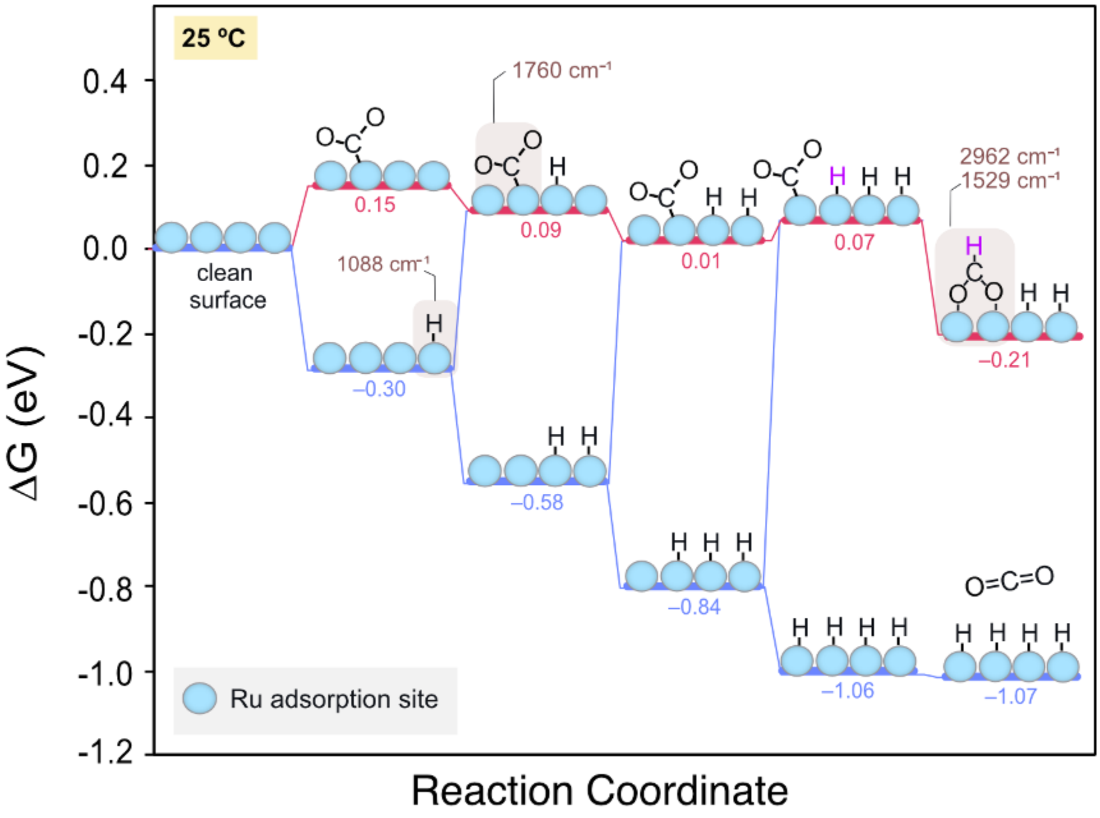

Figure S8. Gibbs adsorption energies of the optimized structures for CO_2 adsorption at different H-coverages on Ru(0001). The calculated Gibbs energies for H adsorption (blue trace) and the different CO_2 and H intermediates (red trace) at room temperature are shown relative to the clean surface.

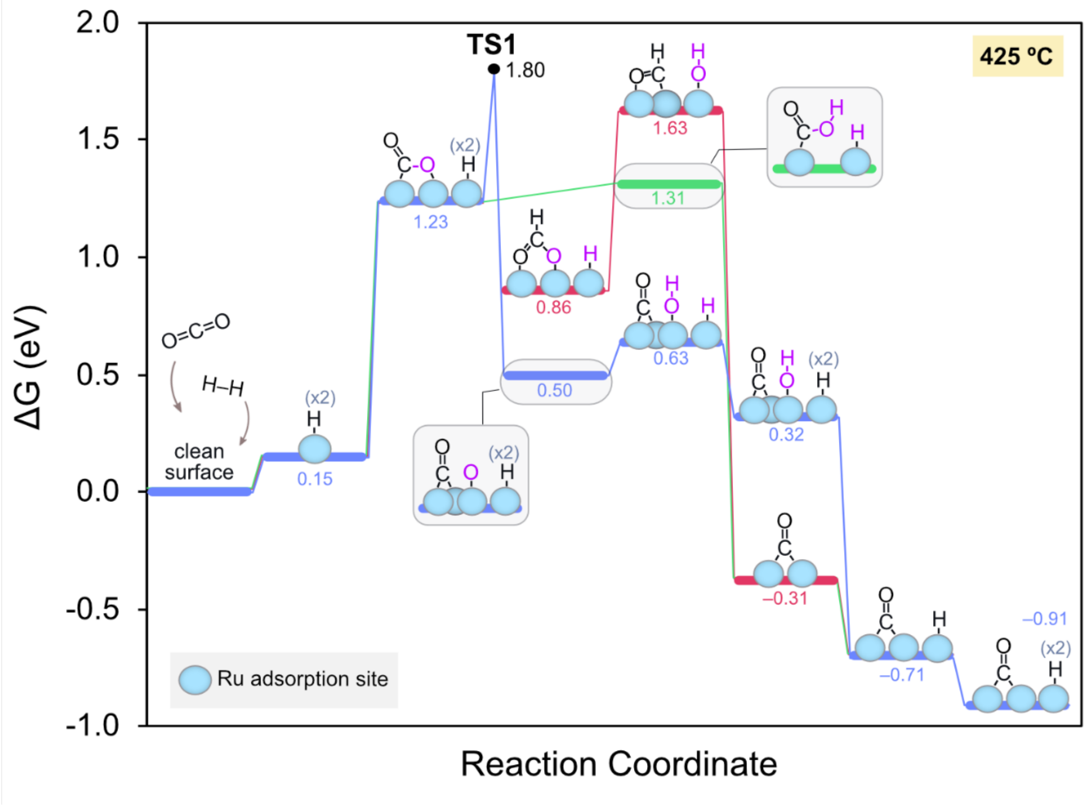

Figure S9. Calculated Gibbs energy diagram for the formation of carbonyl groups on Ru(0001) under CO_2 methanation conditions at 425 ºC.

.

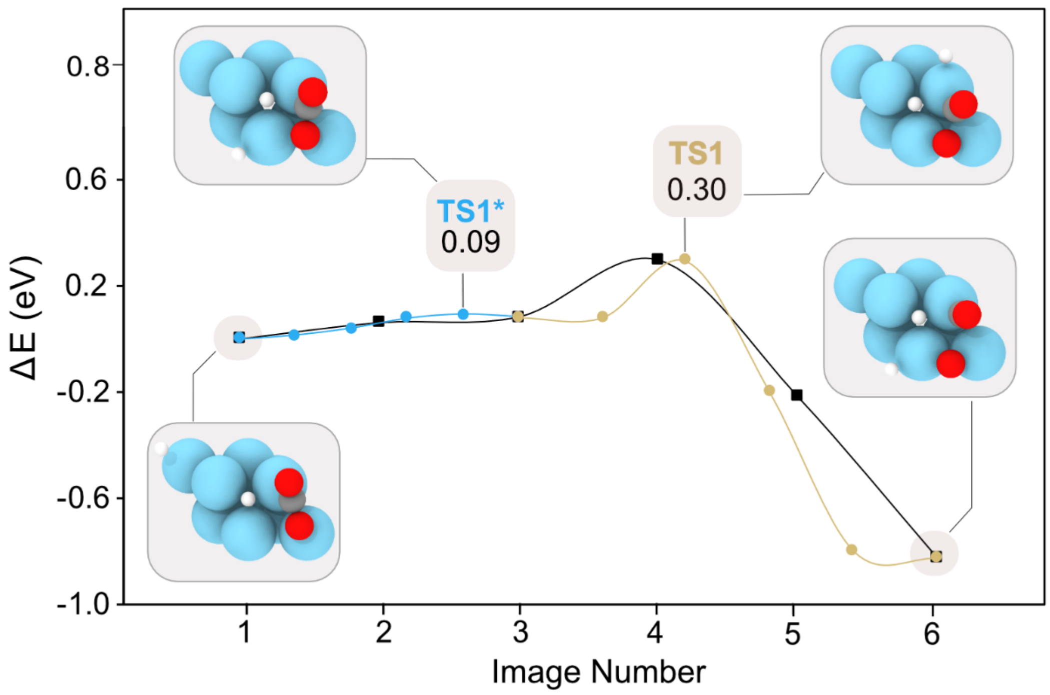

Figure S10. CI-NEB analysis of CO_2 splitting on the Ru(0001) surface with 2 adsorbed H atoms. Insets: top view of the optimized structures of relevant images along the reaction coordinate. CO_2 activation on Ru(0001) proceeds via CO_2 rotation with an activation energy of 0.10 eV, followed by CO_2 dissociation into CO and O with an activation energy of 0.30 eV

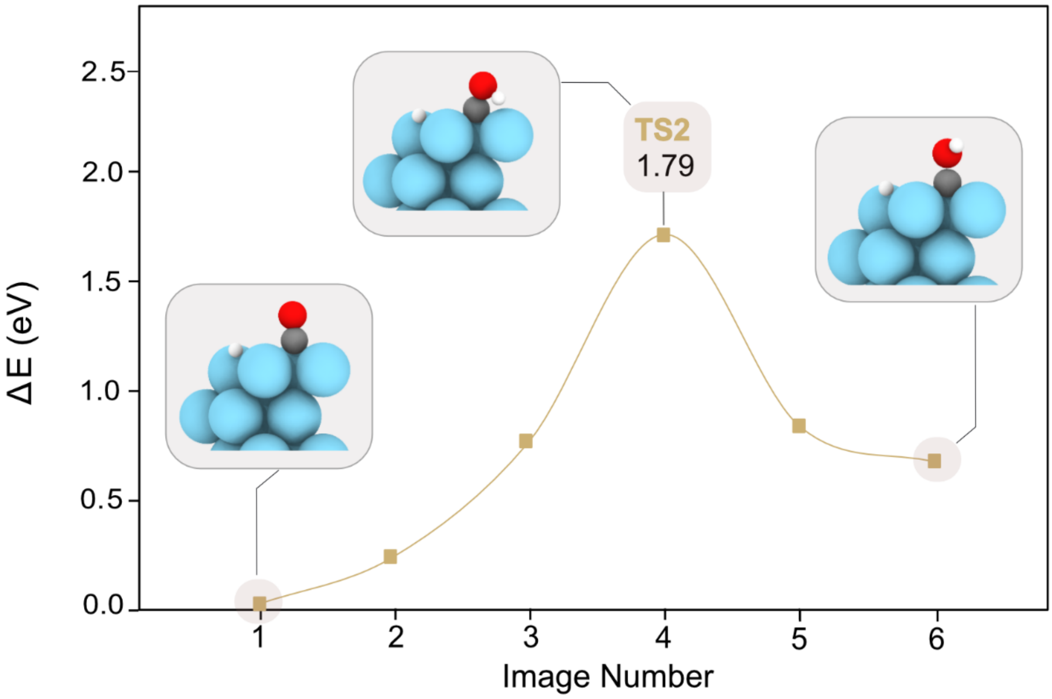

Figure S11. CI-NEB analysis of the CO hydrogenation to COH (step I to IIB in Figure 8) on the Ru(0001) surface.

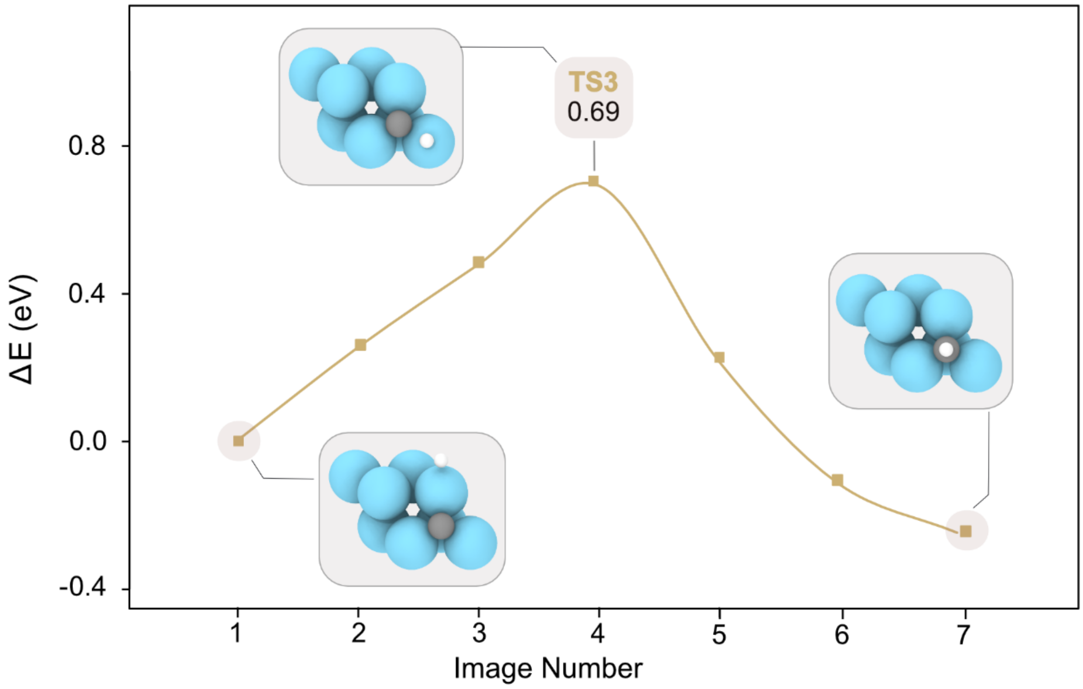

Figure S12. CI-NEB analysis of the hydrogenation of *C to *CH (step IVB to V in Figure 8) on the Ru(0001) surface.

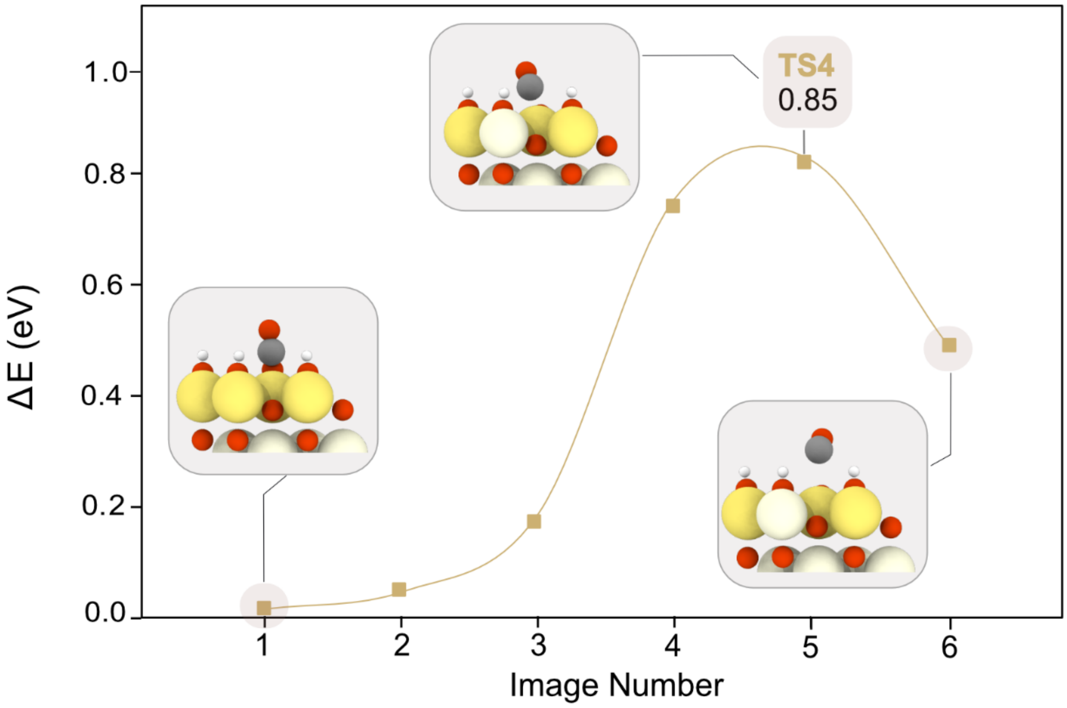

Figure S13. CI-NEB analysis of the dissociation of a carboxylate species into CO and *OH on the $CeO_{2-x}(111)$ surface with 1O vacancy. Color code: Ce^{4+} (pale yellow), Ce^{3+} (yellow).

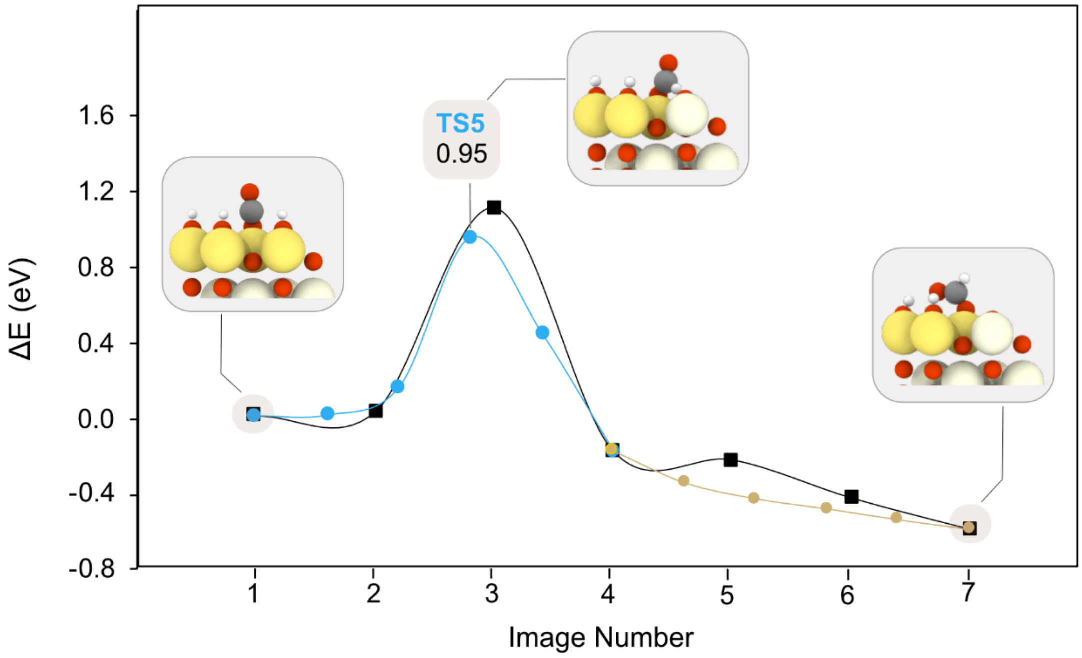

Figure S14. CI-NEB analysis of formate (CHOO*) formation from carboxylate on the $CeO_{2-x}(111)$ surface with 1O vacancy. Color code: Ce^{4+} (pale yellow), Ce^{3+} (yellow).

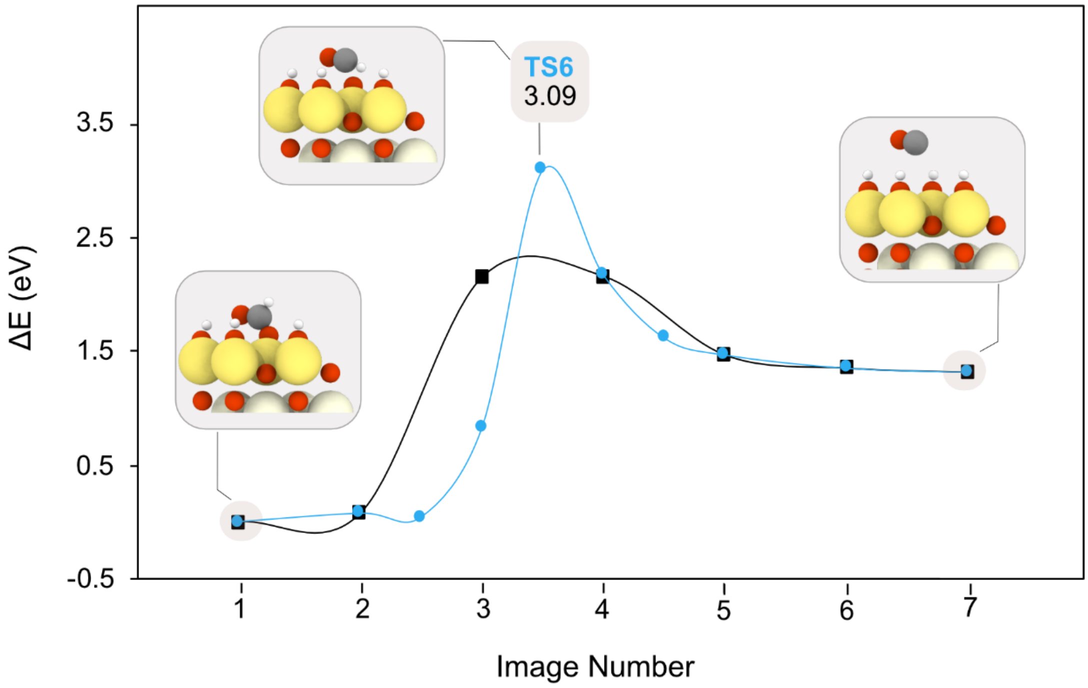

Figure S15. CI-NEB analysis of formate (CHOO*) evolution to *OH and CO release on the CeO_{2-x}(111) surface with 1O vacancy. Color code: Ce^{4+} (pale yellow), Ce^{3+} (yellow).

References

(1) S. Letichevsky, C.A. Tellez, R.R. deAvillez, M.I.P. daSilva, M.A. Fraga, L. G. Appel. Appl. Catal. B **2005,** *58(3–4)*, 203.

(2) S. Tanuma, C. J. Powell, D. R. Penn, *Surf. Interface Anal.* **1994,** *21,* 165; calculated using QUASES-IMFP-TPP2M software, version 3.0; S. Tougaard **2016.**

(3) P. Burroughs, A. Hamnett, A. F. Orchard, G.Thornton, *J. Chem. Soc., Dalton Trans* **1976,** *17,* 1686.
